# Differential regulation of p62-ubiquitin conjugates in neurons versus astrocytes during cellular stress

**DOI:** 10.1371/journal.pone.0345890

**Published:** 2026-04-20

**Authors:** David K. Sidibe, Erin M. Smith, Maeve L. Spivey, Maria C. Vogel, Sandra Maday

**Affiliations:** Department of Neuroscience, Perelman School of Medicine at the University of Pennsylvania, Philadelphia, Pennsylvania, United States of America; Nathan S Kline Institute, UNITED STATES OF AMERICA

## Abstract

Sequestosome 1/p62 (hereafter referred to as p62) is a multifunctional protein that orchestrates various cellular stress response pathways including autophagy, proteasome-mediated degradation, antioxidant defense, nutrient sensing, and inflammatory signaling. Mutations in distinct functional domains of p62 are linked with the neurodegenerative disease amyotrophic lateral sclerosis (ALS), underscoring its importance in neural cells. Neurons and astrocytes, two key cell types in the brain, perform distinct roles in brain physiology and thus encounter a unique landscape of cellular stress. However, how p62 is regulated in these cell types in response to various stress modalities remains largely unexplored. Several functions for p62 depend on its engagement with ubiquitinated substrates. Thus, we investigated how the regulation of p62-ubiquitin conjugates differs between neurons and astrocytes exposed to two stress modalities: lysosomal membrane damage and metabolic stress. Lysosomal damage triggered ubiquitin-dependent assembly of p62 puncta in both neurons and astrocytes. In contrast, nutrient deprivation elicited different responses between neurons and astrocytes. Neurons formed p62-ubiquitin structures more prominently and displayed a greater dependence on ubiquitin for p62 clustering. Together, these findings reveal cell-type-specific and stress-specific regulation of p62-ubiquitin conjugates, indicating that neurons and astrocytes can deploy distinct quality control strategies.

## Introduction

Sequestosome 1 or p62 (hereafter referred to as p62) is a multifunctional protein that is extensively studied for its role as a receptor for selective autophagy [1–3]. P62 binds ubiquitinated substrates via its ubiquitin-associated domain (e.g., UBA domain) and can bridge cargoes with the autophagy machinery through its LC3-interacting region (e.g., LIR) [1]. P62 also has the capacity to self-oligomerize and assemble into phase-separated condensates, which may facilitate many of the cellular functions of p62 [4–8]. With these properties, p62 promotes autophagic clearance of protein aggregates and organelles to maintain cellular homeostasis [1–3,9–12].

Beyond canonical roles in autophagy, p62 also plays important roles in the ubiquitin-proteasome pathway [13,14], anti-oxidant signaling [15–17], pro-inflammatory signaling [18–20], nutrient sensing [21–23], and apoptosis [1]. Thus, p62 integrates protein and organelle quality control and stress response pathways. Many of these functions rely on p62 engaging ubiquitinated substrates to be routed for degradation via autophagosomes [1–3,24–26]. The critical importance of p62 is evidenced by mutations in p62 that are associated with diseases that manifest differently across tissues, including the neurodegenerative disorder amyotrophic lateral sclerosis (ALS) [27–29] and the degenerative bone disease, Paget’s disease [30,31]. Despite extensive study of p62 in immortalized cell lines, the mechanistic diversity of p62 pathway engagement in key cell types of the brain, such as neurons and astrocytes, remains poorly understood.

Neurons and astrocytes perform distinct functions in the brain. Neurons are specialized for rapid synaptic transmission and astrocytes regulate synaptic function, provide metabolic support and serve key roles in buffering oxidative insults and coordinating immune responses. Through these distinct roles in brain physiology, each cell type encounters a unique landscape of cellular stress, which may necessitate different adaptations within quality control and stress response pathways. Indeed, we have shown that metabolic stress and proteotoxic stress induced by proteasomal inhibition elicits differential responses by the autophagy-lysosomal pathway in neurons versus astrocytes [32,33]. Moreover, Rhoads et al. demonstrated that neurons and astrocytes display distinct organelle signatures, encompassing organelle density, dynamics, and inter-organelle contacts, that shift in a stress-dependent manner [34]. However, how p62 may integrate these divergent stress responses in a cell-type-specific manner remains an open question.

Here, we employ a neuron-astrocyte coculture platform to define stress- and cell-type-specific regulation of p62 and its engagement with ubiquitinated substrates. We focus on two stress paradigms that induce quality control pathways previously linked with p62: lysosomal membrane damage and nutrient deprivation. We find that lysosomal membrane damage elicits a robust, ubiquitin-dependent assembly of p62 puncta in both neurons and astrocytes. By contrast, metabolic stress induced by nutrient deprivation stimulates the formation of p62-ubiquitin structures preferentially in neurons as compared with astrocytes. Furthermore, pharmacological reduction in ubiquitination selectively impairs p62 clustering during starvation in neurons as compared with astrocytes. These findings indicate that neurons and astrocytes differentially regulate p62-ubiquitin conjugates in a stress-dependent manner, revealing a layer of mechanistic diversity in how neural cell types engage quality control pathways.

## Materials and methods

### Reagents

**Fig 1:**
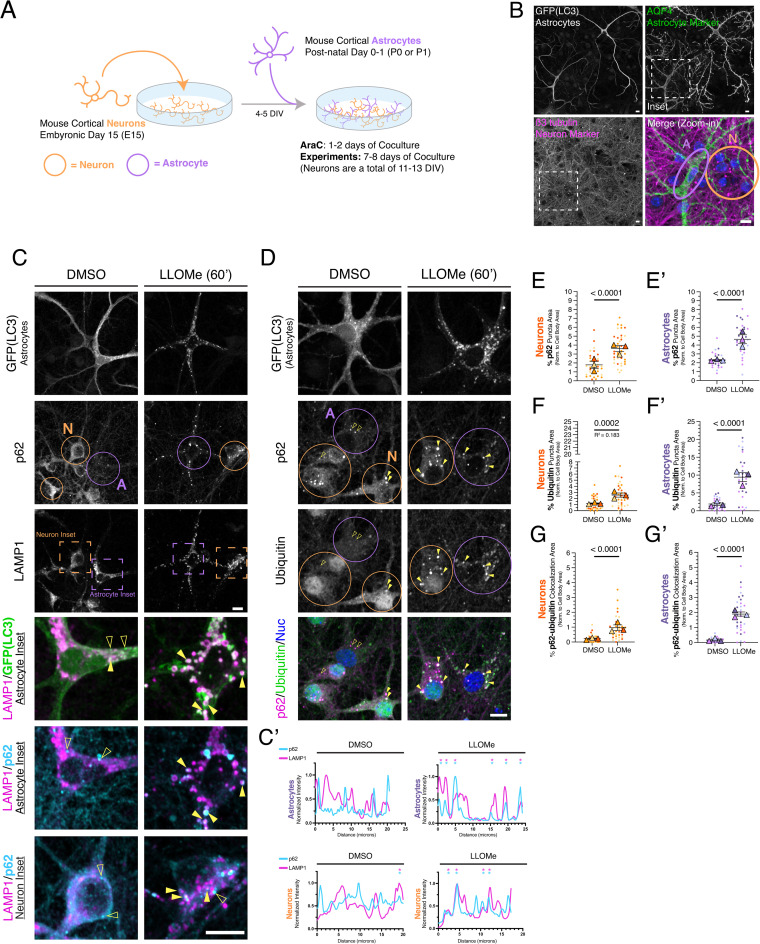
Lysosomal damage triggers the formation of p62-ubiquitin structures in both neurons and astrocytes. **(A)** Schematic of the neuron-astrocyte coculture**. (B)** Representative images of the neuron-astrocyte coculture. Coculture of GFP-LC3 transgenic astrocytes and non-transgenic neurons were fixed on DIV7 of coculture (neurons were a total of DIV11) and immunostained for GFP (LC3; labels GFP-LC3 transgenic astrocytes), AQP4 (astrocyte marker) and β3-tubulin (neuron marker); nuclei were labeled with Hoechst (shown in blue in the merged image). Shown are maximum projections of z-stacks. Dashed box indicates location of the zoom-in. Scale bars, 10 µm. **(C)** Cocultured neurons and astrocytes were treated for 1 hr with 1 mM LLOMe to induce lysosomal damage or an equivalent volume of DMSO solvent as a control. Cocultures were immunostained for GFP (LC3; labels GFP-LC3 transgenic astrocytes), p62, and LAMP1. Shown are maximum projections of z-stacks. Individual images are shown in grayscale; images for GFP(LC3), p62, and LAMP1 are grayscale-matched within a protein marker, across treatment conditions. Filled yellow arrowheads indicate puncta co-positive for GFP(LC3) and LAMP1, or p62 and LAMP1. Empty yellow arrowheads indicate GFP(LC3) or p62 puncta that are negative for LAMP1. Scale bars, 10 µm. (**C’**) Representative line scans of p62 and LAMP1-positive puncta in the soma of neurons and astrocytes. Asterisks denote peaks co-positive for p62 and LAMP1. **(D)** Cocultured neurons and astrocytes were treated for 1 hr with 1 mM LLOMe to induce lysosomal damage or an equivalent volume of DMSO solvent as a control. Cocultures were immunostained for GFP (LC3; labels GFP-LC3 transgenic astrocytes), p62, and ubiquitin; nuclei (Nuc) were labeled with Hoechst. Shown are maximum projections of z-stacks. Individual images are shown in grayscale; images for p62 and ubiquitin are grayscale-matched within a protein marker, across treatment conditions. Filled yellow arrowheads indicate colocalization between p62 puncta and ubiquitin puncta. Empty yellow arrowheads indicate p62 puncta with no ubiquitin puncta correlate. Scale bar, 10 µm. **(E-E’)** Quantification of the total area occupied by p62-positive puncta normalized to soma area for neurons **(E)** or astrocytes **(E’)**. Horizontal bars represent the means of the biological replicates ± SEM; shown are p-values from a LME model; N = 37-40 neurons and N = 27-33 astrocytes from 3 independent experiments; 7-8 DIV of coculture. **(F-F’)** Quantification of the total area occupied by ubiquitin-positive puncta normalized to soma area for neurons **(F)** or astrocytes **(F’)**. Horizontal bars represent the means of the biological replicates ± SEM; shown are p-values from a LME model; N = 35-36 neurons and N = 29-33 astrocytes from 3 independent experiments; 7-8 DIV of coculture. **(G-G’)** Quantification of the percentage of overlapping area between p62-positive puncta and ubiquitin-positive puncta normalized to soma area for neurons **(G)** or astrocytes **(G’)**. Horizontal bars represent the means of the biological replicates ± SEM; shown are p-values from a LME model; N = 32-33 neurons and N = 23-30 astrocytes from 3 independent experiments; 7-8 DIV of coculture. Throughout the figure, neurons are circled in orange and astrocytes are circled in purple. For all graphs in all figures, small circles indicate the measurements from individual cells (e.g., the technical replicates) from each of the independent experiments; large triangles indicate the corresponding biological means from each of the independent experiments (e.g., the biological replicates); independent experiments are color-coded.

**Fig 2:**
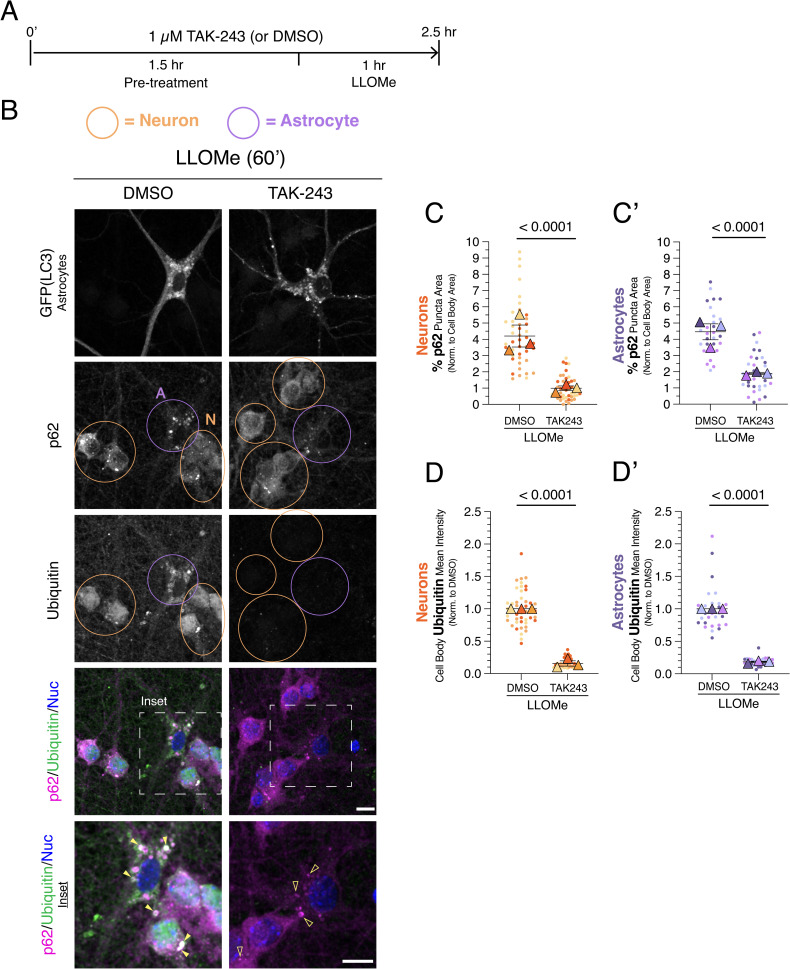
Reducing ubiquitination decreases p62-positive puncta formation during lysosomal damage in both neurons and astrocytes. **(A)** Timeline for experimental treatments. Cocultures of neurons and astrocytes were pretreated for 1.5 hr with 1 µM TAK-243 to inhibit the E1 ubiquitin-activating enzyme or an equivalent volume of DMSO solvent as a control. Cells were then incubated for 1 hour in 1 mM LLOMe to induce lysosome damage, in the continued presence of 1 µM TAK-243 or DMSO. **(B)** Immunostain analysis of co-cultured neurons and astrocytes for GFP (LC3; labels GFP-LC3 transgenic astrocytes), p62, and ubiquitin; nuclei (Nuc) were labeled with Hoechst. Shown are maximum projections of z-stacks. Individual images are shown in grayscale; images for p62 and ubiquitin are grayscale-matched within a protein marker, across treatment conditions. Filled yellow arrowheads indicate colocalization between p62 puncta and ubiquitin puncta. Empty yellow arrowheads indicate p62 puncta with no ubiquitin puncta correlate. Neurons are circled in orange and astrocytes are circled in purple. Scale bars, 10 µm. **(C-C’)** Corresponding quantification of total area occupied by p62-positive puncta normalized to soma area for neurons **(C)** or astrocytes **(C’)**. Horizontal bars represent the means of the biological replicates ± SEM; shown are p-values from a LME model; N = 46-47 neurons and N = 29-33 astrocytes from 3 independent experiments; 7-8 DIV of coculture. **(D-D’)** Quantification of the normalized mean intensity of ubiquitin signal in the cell body of neurons **(D)** or astrocytes **(D’)**. Horizontal bars represent the means of the biological replicates ± SEM; shown are p-values from a LME model; N = 44-45 neurons and N = 29-34 astrocytes from 3 independent experiments; 7-8 DIV of coculture.

**Fig 3:**
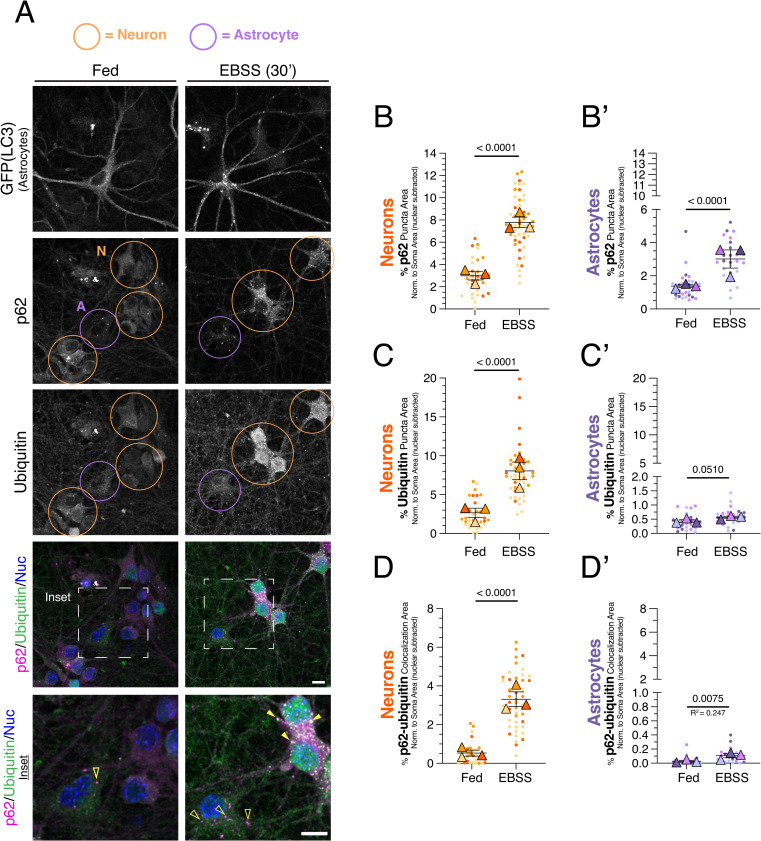
Short-term metabolic stress preferentially increases p62-ubiquitin structures in neurons as compared to astrocytes. **(A)** Cocultures of neurons and astrocytes were treated for 30 minutes in EBSS (or regular media as a fed control) and then immunostained for GFP (LC3; labels GFP-LC3 transgenic astrocytes), p62, and ubiquitin; nuclei (Nuc) were labeled with Hoechst. Shown are maximum projections of z-stacks. Individual images are shown in grayscale. Filled yellow arrowheads indicate colocalization between p62 puncta and ubiquitin puncta. Empty yellow arrowheads indicate p62 puncta with no ubiquitin puncta correlate. Neurons are circled in orange and astrocytes are circled in purple. Scale bars, 10 µm. **(B-B’)** Corresponding quantification of total area occupied by p62-positive puncta normalized to cytoplasmic area in the soma for neurons **(B)** or astrocytes **(B’)**. Horizontal bars represent the means of the biological replicates ± SEM; shown are p-values from a LME model; N = 34-52 neurons and N = 27-31 astrocytes from 3 independent experiments; 7 DIV of coculture. **(C-C’)** Corresponding quantification of area occupied by ubiquitin-positive puncta normalized to cytoplasmic area in the soma for neurons **(C)** or astrocytes **(C’)**. Horizontal bars represent the means of the biological replicates ± SEM; shown are p-values from a LME model; N = 31-48 neurons and N = 24-25 astrocytes from 3 independent experiments; 7 DIV of coculture. **(D-D’)** Quantification of the percentage of overlapping area between p62-positive puncta and ubiquitin-positive puncta normalized to cytoplasmic soma area for neurons **(D)** or astrocytes **(D’)**. Horizontal bars represent the means of the biological replicates ± SEM; shown are p-values from a LME model; N = 29-48 neurons and N = 22-23 astrocytes from 3 independent experiments; 7 DIV of coculture.

**Fig 4:**
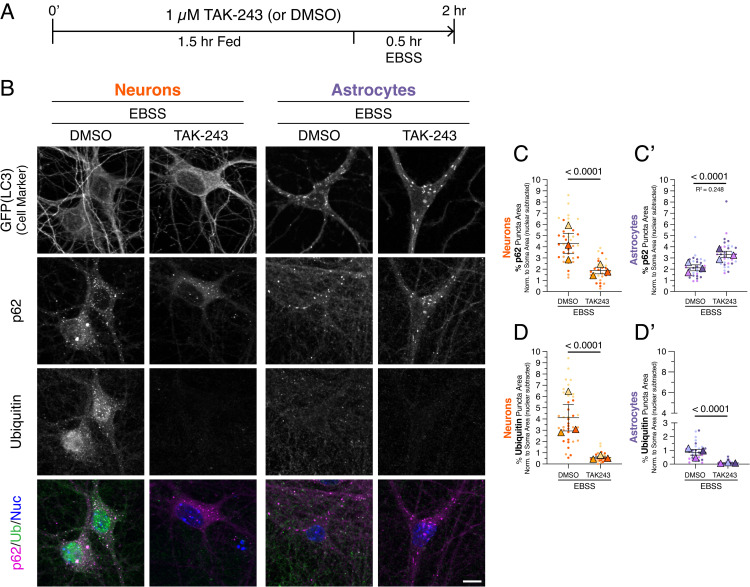
Reducing ubiquitination decreases p62 puncta formation during metabolic stress in neurons but not in astrocytes. **(A)** Timeline for experimental treatments. Cocultures of neurons and astrocytes were pretreated for 1.5 hr with 1 µM TAK-243 to inhibit the E1 ubiquitin-activating enzyme or an equivalent volume of DMSO solvent as a control. Cells were then incubated for 30 minutes in EBSS to induce nutrient deprivation, in the continued presence of 1 µM TAK-243 or DMSO. **(B)** Immunostain analysis of cocultured neurons and astrocytes for GFP(LC3), p62, and ubiquitin; shown are maximum projections of z-stacks. Individual images are shown in grayscale. To identify neurons, GFP-LC3 transgenic neurons were cocultured with non-transgenic astrocytes. Since lipidated GFP-LC3 is known to not preserve well during fixation specifically in neurons, the GFP-LC3 transgene serves only as a cell-type-specific marker to identify neurons. To identify astrocytes, GFP-LC3 transgenic astrocytes were cocultured with non-transgenic neurons. Nuclei (Nuc) were labeled with Hoechst. Scale bar, 10 µm. **(C-C’)** Quantitation of total area occupied by p62-positive puncta normalized to cytoplasmic soma area for neurons **(C)** or astrocytes **(C’)**. Horizontal bars represent the means of the biological replicates ± SEM; shown are p-values from a LME model; N = 30-39 neurons and N = 32-34 astrocytes from 3 independent experiments; 7-8 DIV of coculture. **(D-D’)** Quantitation of total area occupied by ubiquitin-positive puncta normalized to cytoplasmic soma area for neurons **(D)** or astrocytes **(D’)**. Horizontal bars represent the means of the biological replicates ± SEM; shown are p-values from a LME model; N = 30-38 neurons and N = 32-34 astrocytes from 3 independent experiments; 7-8 DIV of coculture.

Primary antibodies for immunofluorescence include mouse anti-βIII Tubulin (R&D Systems, MAB1195), rabbit anti-AQP4 (Millipore Sigma, HPA014784), chicken anti-GFP (Aves Labs, Inc., GFP-1020), rabbit anti-p62 (Abcam, Ab109012), mouse anti-Ubiquitin (Enzo Life Sciences, ENZ-ABS840; Figs 1−3 and S3), mouse anti-Ubiquitin (Enzo Life Sciences, BML-PW8810; Figs 3, 4 and S3), and rat anti-LAMP1 (Abcam, ab25245). We used two ubiquitin antibodies because BML-PW8810 was discontinued during the course of this study, and was replaced with ENZ-ABS840. We previously validated both antibodies to specifically recognize ubiquitin [33]. Hoechst 33342 was purchased from Thermo Fisher Scientific/Molecular Probes (H3570). Secondary antibodies for immunofluorescence include goat anti-chicken Alexa Fluor 488 (Jackson ImmunoResearch Laboratories, 103-545-155), goat anti-rabbit Alexa Fluor 594 (Invitrogen, A11037), goat anti-mouse Alexa Fluor 647 (Invitrogen, A32728), goat anti-rat Alexa Fluor 647 (Invitrogen, A21247), goat anti-mouse Alexa Fluor 594 (A11032; Fig 2), and goat anti-rabbit Alexa Fluor 647 (A21245; Fig 2). Small-molecules include L-Leucyl-L-Leucine methyl ester (LLOMe; Cayman Chemicals, CAYM-16008) and TAK-243 (MLN7243; SelleckChem, S8341); small molecules were dissolved in DMSO (Sigma, 472301). Earle’s balanced salt solution (EBSS) was purchased from Sigma (E3024).

### Coculture of primary mouse cortical neurons and cortical astrocytes

#### Primary cortical neuron culture.

Transgenic mice expressing GFP-LC3 (EGFP fused to the N-terminus of rat LC3B) were obtained from the RIKEN BioResource Research Center (RBRC00806; strain B6.Cg-Tg(CAG-EGFP/LC3)53 Nmi/NmiRbrc; GFP-LC3#53) and maintained as heterozygotes. The GFP-LC3 transgene is widely expressed using a constitutive CAG composite promoter that combines a cytomegalovirus immediate early enhancer, a chicken β-actin gene promoter and a rabbit β-globin splice acceptor [35]. All animal protocols were approved by the Institutional Animal Care and Use Committee at the University of Pennsylvania (Protocol #805934). For procedures isolating embryos, timed-pregnant females were euthanized at embryonic day 15.5 (E15) by overdose of isoflurane; euthanasia of the mother was confirmed by cervical dislocation and euthanasia of the embryos was confirmed by decapitation. Cerebral cortices were dissected from brains of GFP-LC3 transgenic mouse embryos (or non-transgenic littermates) of either sex at embryonic day 15.5. GFP-LC3 transgenic brain tissue was distinguished from non-transgenic brain tissue at the start of the dissection based on the presence or absence of GFP fluorescence, respectively. Tissue was then digested and cortical neurons were isolated following our published protocols [32,36]. Neurons were plated at 3 million cells per 10-cm dish containing eight 25-mm acid-washed glass coverslips, or 1.15 million cells per 6-cm dish containing twelve 12-mm coverslips or five 18-mm coverslips (plating density of ~49,000 cells/cm^2^), coated with 0.1 mg/mL poly-L-lysine (Sigma, P2636). When GFP-LC3 transgenic neurons were used, as in Fig 4, they were diluted 1:10 with non-transgenic neurons and plated onto 10 cm-dishes containing 8 x 25 mm coverslips at the density described above. Neurons were plated in attachment media (MEM [11095–072] supplemented with 10% (v/v) heat-inactivated horse serum [Gibco, 16050–122], 1 mM sodium pyruvate [Gibco, 11306–070], 33 mM glucose [Sigma,G8769], and 37.5 mM NaCl) and incubated at 37°C in a 5% CO_2_ incubator for 1–4 hours. Neurons were then transferred into either 12-well dishes (for 12-mm coverslips) or 6-well dishes (for 18-mm or 25-mm coverslips) and maintained for 4–5 DIV in neuron maintenance media (Neurobasal medium [Gibco, 21103–049] supplemented with 2% B-27 [Gibco,17504–044], 37.5 mM NaCl, 33 mM glucose [Sigma,G8769], 2 mM glutaMAX, and 100 U/ml penicillin and 100 μg/ml streptomycin) at 37°C in a 5% CO_2_ incubator before the addition of astrocytes (discussed below). In some experiments in Figs 3 and S3, neurons were fed on 2–3 DIV of the neuron culture, where 25% media was replaced, and 1 μM AraC (antimitotic drug; Sigma, C6645) was added to reduce glial growth.

#### Primary cortical astrocyte culture.

Cerebral cortices were dissected from brains of GFP-LC3 transgenic neonatal mice (or non-transgenic littermates) of either sex at post-natal day 0 or 1 (P0-P1); mouse neonates at stage P0-P1 were euthanized by decapitation. GFP-LC3 transgenic brain tissue was distinguished from non-transgenic brain tissue at the start of the dissection based on the presence or absence of GFP fluorescence, respectively. Tissue was then digested and cortical glia were isolated following our published protocols that yield glial preparations that are highly enriched for astrocytes [32,36]. Glia were plated at 1−3 million cells per 10-cm dish and cultured in glial media (DMEM [Gibco, 11965–084] supplemented with 10% (v/v) heat inactivated fetal bovine serum [Hyclone, SH30071.03], 2 mM Glutamax [Gibco, 35050–061], 100 U/ml penicillin and 100 μg/ml streptomycin [Gibco, 15140–122]) at 37°C in a 5% CO_2_ incubator. The day after plating, and every 3−4 days following, glia were fed by fully replacing the glial media. When glia reached 70−80% confluency, astrocytes were dissociated from the 10 cm tissue culture dish using 0.05% trypsin-EDTA (Gibco, 15400−054) and plated onto neurons. Occasionally, to coordinate timing with the neuron preparation, astrocytes were passaged an additional time before plating onto neurons. To reduce microglial contamination, glial culture dishes were vigorously tapped to dislodge microglia before aspirating and discarding media when feeding or passaging cells.

#### Coculture of primary cortical neurons and astrocytes.

On 4–5 DIV of neuronal culture, astrocytes were trypsinized from the 10-cm plate, as described above, and used for coculture with neurons. After dissociation from the 10-cm tissue culture dish, trypsin was inactivated using glial media. Astrocytes were then spun down at 644 x g for 2 minutes and resuspended in coculture media (Neurobasal supplemented with 2% B-27, 1% G-5 [Gibco, 17503–012], 0.25% GlutaMAX, 100 U/ml penicillin, and 100 µg/ml streptomycin); the G5 supplement promotes astrocyte growth and maturation. Neuronal maintenance media was removed from the wells containing neurons, and astrocytes were plated onto the neurons at a density of 5,200 or 7,000 cells/cm^2^ (depending on glial growth rate prior to glial passaging) in coculture media, totaling 50,000 or 67,500 astrocytes per 6-well, or 20,000 or 25,000 glia per 12-well, respectively. The coculture was maintained at 37°C in a 5% CO_2_ incubator. After 1–2 DIV of coculture, AraC was added to the cells in 100 µl of coculture medium to reach a final concentration of 2 μM AraC to prevent cell division and promote maturation of the astrocytes. The coculture was then maintained in the incubator until experiments were performed on 7–8 DIV of coculture (11–13 DIV of neuron culture) (Figs 1A and S1).

### Stress treatments

#### Lysosomal damage.

Cocultures were treated with 1 mM L-Leucyl-L-Leucine methyl ester (i.e., LLOMe) for 1 hr at 37°C in a 5% CO_2_ incubator. To perform the treatment, conditioned coculture media (i.e., media already present on the cells) was diluted 1:1 with fresh coculture media (pre-warmed to 37°C), and supplemented with 1 mM LLOMe or an equivalent volume of DMSO as a solvent control (DMSO volume is 0.4% of total treatment volume).

#### Starvation (metabolic stress) treatment.

Coculture media was replaced with either Earle’s Balanced Salt Solution (EBSS, Sigma, E3024) to starve the cells, or replaced with fresh coculture media (fed controls); both medias were pre-warmed to 37°C in a water bath prior to treatment. Treatments contained 0.1% DMSO. For short-term treatments, samples were incubated for 30 minutes at 37°C in a 5% CO_2_ incubator. For long-term treatments, samples were incubated for 4 hours at 37°C in a 5% CO_2_ incubator.

#### Inhibition of the E1 ubiquitin-activating enzyme.

Cocultures were pretreated for 1.5 hr with 1 µM TAK-243 (MLN7243) or an equivalent volume of DMSO as a solvent control at 37°C in a 5% CO_2_ incubator. To perform the pretreatment, conditioned coculture media was diluted 1:1 with fresh coculture media (pre-warmed at 37°C in a water bath), and supplemented with 1 µM TAK-243 or an equivalent volume of DMSO as a solvent control (DMSO volume is 0.1% of total treatment volume); samples were incubated for 1.5 hr at 37°C in a 5% CO_2_ incubator. For Fig 2, after the 1.5-hour pretreatment, the pretreatment media was diluted 1:1 with fresh coculture media (pre-warmed at 37°C in a water bath), and supplemented with 1 mM LLOMe (or equivalent volume of DMSO) and TAK-243 (or equivalent volume of DMSO) to a final concentration of 1 µM TAK-243 (total DMSO volume is 0.5% of total treatment volume). Samples were incubated for 1 hr at 37°C in a 5% CO_2_ incubator (Fig 2A). For Fig 4, after the 1.5 hr TAK-243 pretreatment, all treatment media was removed and replaced with EBSS supplemented with 1 µM TAK-243 (or DMSO as a solvent control; total DMSO volume is 0.1% of total treatment volume); samples were incubated for 30 min at 37°C in a 5% CO_2_ incubator.

### Immunostaining

Following treatments, cocultured cells were fixed for 10 min in 4% PFA/4% sucrose (PFA, Sigma, P6148; D-sucrose, Fisher Scientific, BP220−1) in 1X PBS (150 mM NaCl, 50 mM NaPO_4_, pH 7.4); fixative was pre-warmed to 37°C prior to cell fixation. Cells were washed three times in 1X PBS, and stored in PBS at 4°C for 1−2 days until subsequent processing. Cells were then permeabilized for 5 min in 0.1% Triton X-100 (Thermo Fisher Scientific, BP151−100) in 1X PBS, washed twice with 1X PBS, and blocked for 1 hr in 1X PBS supplemented with 5% (v/v) goat serum (Sigma, G9023) and 1% (w/v) BSA (Thermo Fisher Scientific, BP1605−100). Samples were labeled with primary antibodies diluted in blocking solution for 1 hr at room temperature. Cells were washed 3 times for 5 min each in 1X PBS, and then incubated in secondary antibody diluted in blocking solution for 1 hr at room temperature. Coculture samples were washed 3 times for 5 min each in 1X PBS, washed once in Milli-Q water, and mounted on glass microscope slides (ThermoFisher 12-544-2) in ProLong Gold (Thermo Fisher Scientific/Molecular Probes, P36930). When indicated in the figure, Hoechst (Thermo Fisher Scientific/Molecular Probes, H3570) was included at 0.5 μg/ml either during the secondary antibody incubation or in the final PBS wash before mounting coverslips; Hoechst addition at either step yielded comparable fluorescence intensity. We note that fixation can quench or attenuate the GFP signal from the GFP-LC3 transgene. Therefore, immunostaining against GFP was performed to enhance signal detection. All steps of the immunostain procedure were performed at room temperature with the samples protected from ambient light.

Samples were imaged on a BioVision spinning disk confocal system consisting of a Yokagawa W1 spinning disk confocal and a Photometrics Prime 95B sCMOS camera. Images were acquired with VisiView software using either a 40X/1.4 NA (Fig 1B) or 63X/1.4 NA (all other figures) Plan-Apochromat oil-immersion objective with either a 1X (Figs 1–3, S2A–S2E, S3 and S4) or 2X (Figs 4, S2F and S2G) optical magnifier and solid-state 405, 488, 561, and 640 nm lasers for excitation. Z-stacks were obtained that spanned the entire depth of the cell at 0.2-μm sections. Images that would be quantitatively compared to each other were obtained with the same acquisition parameters across treatment conditions and biological replicates.

### Image analysis

#### Linescan analysis of p62 and LAMP1.

We first generated max projections of Z-stacks in FIJI. Linescans were drawn from representative images in Fig 1C. To create the linescans, lines were drawn through puncta in the p62 channel and the same line was overlaid on the LAMP1 channel. The plot profile function in FIJI was used to quantify gray values along the line in each channel. Each channel was then normalized to its own maximum. Both p62 and LAMP1 normalized linescans from the same cell type and treatment condition were plotted on the same graph, and events where p62 and LAMP1 puncta overlapped were denoted with asterisks.

#### Area occupied by GFP(LC3), p62, and ubiquitin positive puncta.

We first generated maximum projections of Z-stacks in FIJI. Astrocytes were selected for analysis based on morphological complexity, containing at least 3 well-defined processes that developed secondary and tertiary branches. Neurons were selected for analysis based on the following criteria: neurons must exhibit a well-defined axon and at least 3 well-defined and branching dendrites, one of which being a primary dendrite. For Figs 4 and S4, astrocyte and neuronal somas were outlined using the GFP(LC3) channel and measured for total cross-sectional area; the ROIs outlining the somas were saved for subsequent use. For all other figures, neurons were selected and outlined in the p62 channel, using the cytosolic levels of p62 as a space fill, and astrocytes were selected and outlined using the GFP(LC3) channel. ROIs outlining the somas were saved for subsequent use. We then removed signal outside the ROI using the FIJI function “clear outside” and the individual cell images were then loaded into Ilastik to identify and segment puncta of interest. To reduce bias in selecting cells for analysis, images were blinded to treatment identity during cell selection using the “Filename Randomizer” plugin (credit: Tiago Ferreira) for the following figures: Figs 3, S2B–S2E and S3. Ilastik was used to identify and segment GFP(LC3)-positive puncta (as measured using the GFP antibody), p62-positive puncta, and ubiquitin-positive puncta. To account for cell-type-specific differences in signal intensity, we trained separate Ilastik programs for each marker across treatment paradigms for each cell type. The only exception is that we used the same Ilastik program for quantifying ubiquitin puncta in astrocytes for both the 30 minutes and 4 hours EBSS treatment paradigms (as in Figs 3C’ and S3C’). To enable direct comparisons, the same Ilastik program was always used for all replicates of a given experiment. Ilastik segmentations were then imported into FIJI to generate a mask of the segmentation. The total area occupied by segmented puncta was then measured using the “analyze particles” function in FIJI. Total area occupied by LC3, p62, and ubiquitin-positive puncta per cell was normalized to the corresponding cell body area (or cytoplasmic area, as described below) and plotted as a percentage of total cell body area. To calculate the average size of puncta (e.g., S4 Fig), the total puncta area was divided by the number of puncta per cell; values were obtained using the “analyze particles” function in FIJI.

#### Nuclear subtraction.

In the starvation treatments, we observed that ubiquitin puncta increased in the nucleus of neurons. This observation may be related to known functions for ubiquitin in regulating proteasomal activity and histone modification to regulate DNA accessibility in certain paradigms of stress [37–40]. Since we were primarily interested in quantifying the population of ubiquitin in the cytoplasm, we removed the nuclear area from the cell body area (Figs 3, 4, S2B–S2G, S3 and S4). To do this, we first trained an Ilastik program to recognize the nucleus based on the Hoechst stain for each outlined cell. We then created a mask of the nuclear signal in FIJI and inverted the mask to create a mask of the cytoplasm. Using the “AND” function in the image calculator tool in FIJI, we counted pixels that were present in both the cytoplasm mask and the mask of the total puncta for each protein of interest. The area of cytoplasmic puncta for each protein was then normalized to the cytoplasmic area for each individual cell and expressed as a percentage.

#### Colocalization area.

To measure colocalization between p62 and GFP(LC3) or ubiquitin puncta, we determined the overlapping area between the Ilastik segmentations for p62, GFP(LC3), and ubiquitin puncta that were generated as described above. Using FIJI, we then measured the overlapping area between indicated markers using the “AND” function in the image calculator tool for each individual cell. For starvation datasets, we used the nuclear-subtracted segmentations for colocalization analysis. Total overlapping area was then normalized to the corresponding soma area (or cytoplasmic area for the nuclear-subtracted analysis) and expressed as a percentage.

#### Soma ubiquitin analysis.

For Fig 2D–D’, we used the ROIs of the soma outlines generated above to measure the ubiquitin signal in the soma. The ROI outlining the soma was overlaid onto the maximum projection of the ubiquitin channel for each individual cell, and we measured the mean gray value of the ubiquitin signal using FIJI. For each condition and biological replicate, we also measured the mean gray value of a circular ROI that was drawn in an area with no cellular processes or meshwork to represent the background signal. This mean gray value for background was then subtracted from the corresponding ubiquitin mean gray value for each individual cell to create a background-corrected mean gray value for ubiquitin (hereafter referred to as “mean gray value”). We then normalized the mean gray values for each individual cell in both the control and treatment groups to the corresponding DMSO control average for each independent experiment and each cell type. Normalized mean gray values for each soma (e.g., technical replicates) were averaged to generate a mean for each independent experiment (e.g., biological replicates).

### Figure and manuscript preparation

For presentation of images, maximum and minimum gray values were adjusted linearly in FIJI. Images that are grayscale matched are denoted in the relevant figure legend. Superplots of graphs were assembled in Graphpad Prism according to published techniques [41]. In all graphs in all figures, small circles indicate the measurements from individual cells (e.g., the technical replicates) from each of the independent experiments; large triangles indicate the corresponding biological means from each of the independent experiments (e.g., the biological replicates); bars are the means of the biological means ± SEM; independent experiments are color-coded. Figures were assembled using Adobe Illustrator. The microscope image in S1 Fig was generated using the “Generate Vector” AI tool in Illustrator. Perplexity and ChatGPT AI tools were used to make some sentences and paragraphs more concise to improve clarity and readability. After use of these tools, the authors carefully reviewed and edited the content as needed and take full responsibility for the content of the publication.

### Statistical analyses

All image measurements were obtained from the raw data. For all datasets, we used RStudio (Version 2025.5.0.496) to run a linear mixed effects model (LME, R package “lme4”). Our data have a nested structure, with multiple cells (technical replicates) measured within each biological replicate (independent cell preparations from different mice) that are exposed to different treatments (fixed effects). To account for the non-independence between the technical replicates within each biological replicate, we fit a linear mixed-effects model with biological replicates defined as a random effect. This model allows us to assess treatment-level effects while preserving the variability of individual cells within the hierarchical structure of the data. To confirm that the model fit was appropriate, we assessed the homoscedasticity of the model (i.e., whether the variance of the data is equal across the entire range of the fixed effect) for each dataset analyzed. In datasets where the model was not homoscedastic, we utilized the “VarIdent” function (R package “nlme”) to standardize the variance between different treatment conditions and improve the LME model fit. We used the “VarIdent” function for the following datasets: Figs 1F’, 1G, 1G’, 2C, 2D, 2D’, 3C, 3D,3D’, 4D, 4D’ S2C–S2E, S3B, S3B’, S3D and S4A. All other datasets used the unadjusted LME model. To confirm that the results of the LME model did not differ between the two LME packages used (lme4 versus nlme), we ran several LME models (without any corrections) using the same data in both packages and found the p-values to be nearly identical. We used marginal R^2^ values to assess how much of the variance was explained by the model. R^2^ values of 0.1–0.25 are considered modest effects, whereas R^2^ values less than 0.1 are considered weak effects. Unless noted directly in the figure, R^2^ values were above a cutoff of 0.25, indicating that the treatment variable (e.g., LLOMe) accounts for a substantial portion of the variance. When an R^2^ value was below 0.25 but statistical significance was achieved, this combination indicates that the treatment variable has a modest effect, but explains only a limited amount of the outcome variance and is noted in the relevant graph. When an R^2^ value was below 0.1 but statistical significance was achieved, this combination indicates that the treatment variable has a weak effect, but explains only a very limited amount of the outcome variance and is noted in the relevant graph. All quantifications were performed with 3 independent experiments, and statistical comparisons were only made within cell type, not between cell type. P-values are denoted directly within each graph.

## Results

### Neurons and astrocytes exhibit similar p62-ubiquitin responses to lysosomal damage

To investigate the regulation of p62-ubiquitin conjugates in neurons and astrocytes in models of cellular stress, we cocultured primary mouse cortical neurons with primary mouse cortical astrocytes. Neurons (~96% pure based on our prior analysis with a neuron-specific marker [32]) are cultured for 4–5 days *in vitro* (DIV) to establish a dense meshwork of neurites before addition of astrocytes (Figs 1A and S1). Astrocytes were then plated on top of the neuron meshwork and cocultured for 7–8 DIV, resulting in a total neuronal age of 11–13 DIV; Figs 1A and S1). This order of addition is critical as direct contact and secreted factors from developed neurons promote astrocyte maturation [42, 43]. In this context, astrocytes develop arborized processes reminiscent of morphologies observed *in vivo* (Fig 1B). Immunostaining for aquaporin 4 (AQP4), an astrocyte-enriched water channel, and β3-tubulin, a marker of neuronal microtubules, reveals the branched morphologies of astrocytes in coculture with a dense neuronal network (Fig 1B).

We also made use of a transgenic mouse expressing GFP-LC3 [35] to distinguish cell-types (S1 Fig). In its cytosolic form, LC3 can delineate cellular morphologies and boundaries and, when lipidated, LC3 labels autophagosome membranes. This tool enables visualization of morphological compartments, quantification of autophagy levels, and validation of stress-inducing treatments. To distinguish astrocytes from neurons, astrocytes isolated from GFP-LC3 transgenic mice were cocultured with non-transgenic neurons (Figs 1B and S1C). Based on our previously published purity analysis for our neuron preparation [32], we classified all non-transgenic cells with neuronal morphology as neurons (see Methods). Together, this neuron-astrocyte coculture provides a method to examine cell-type-specific regulation of p62-ubiquitin conjugates in response to defined stress paradigms.

We first examined cell-type-specific responses to lysosomal membrane damage elicited by L-leucyl-L-leucine methyl ester (LLOMe), a lysosomotropic agent that is processed by cathepsin C to generate membranolytic polymers that permeabilize lysosomal membranes [44]. Sustained exposure to LLOMe promotes the autophagic removal of damaged lysosomes, a process known as lysophagy, which involves the recruitment of selective autophagy receptors, including p62, to ubiquitinated substrates on the lysosome [3,26,45,46]. This pathway has been extensively characterized in immortalized cell lines, but less is known about how neurons and astrocytes respond to lysosomal injury, despite pathophysiological relevance for lysosomal quality control in neurodegenerative disease.

To induce lysosomal membrane damage, cocultures were treated with 1 mM LLOMe for 1 hr; astrocytes were identified by expression of GFP-LC3 (S1C Fig). The effectiveness of the treatment was validated by quantifying GFP-LC3-positive puncta in astrocytes. During early stages of lysosomal damage, LC3, an ATG8 family protein, is directly conjugated to lysosomal membranes in a process termed CASM (conjugation of ATG8 to single membranes), which may support lysosomal repair [47,48]. More prolonged or severe damage leads to LC3 recruitment to eliminate irreparably damaged lysosomes by lysophagy [49]. Thus, the appearance of LC3 on lysosomes, via CASM or lysophagy, serves to support the occurrence of lysosomal damage. To quantify GFP-LC3 puncta, we used Ilastik [50], a machine-learning platform trained to segment punctate structures from diffuse cytosolic signal. LLOMe treatment caused a marked increase in GFP-LC3-positive puncta and ring-like structures in astrocytes relative to the solvent control (Figs 1C and S2A). These LC3 structures overlapped with LAMP1, a marker for late endosomes and lysosomes (Fig 1C; colocalization indicated with filled yellow arrowheads). In a parallel study, we established that LLOMe induces lysosomal damage in astrocytes and neurons by various functional and morphological metrics [51]. Combined, these results confirm that LLOMe efficiently induces lysosomal damage and triggers the formation of LC3-positive structures.

Next, we immunostained neuron-astrocyte cocultures for p62 and ubiquitin. We used two ubiquitin antibodies that detect both mono- and polyubiquitination, including K29-, K48-, and K63-linked species. As validated in our previous work, these antibodies recognize p62-positive fibrils and aggresomes that form in astrocytes under conditions of proteasomal inhibition [33]. In DMSO-treated controls, occasional p62-positive puncta were detected in both neurons and astrocytes (Fig 1D), consistent with basal homeostatic functions for p62. In neurons, ubiquitin localized primarily to the nucleus with variable levels of cytoplasmic puncta (Fig 1D). Nuclear localization is consistent with the established role of ubiquitin in chromatin remodeling [52]. In astrocytes, ubiquitin levels were lower overall and predominantly cytoplasmic (Fig 1D).

LLOMe treatment significantly increased the formation of p62- and ubiquitin-positive puncta in the cytoplasm of both neurons and astrocytes relative to solvent controls (Fig 1D–G’). However, we note that the increase in ubiquitin-positive puncta was more prominent in astrocytes than neurons (Fig 1F–F’). Since degradative lysosomes are known to concentrate in the soma of neurons [53–56], we focused our analysis in this region of neurons and astrocytes. LLOMe exposure also increased colocalization between p62 and ubiquitin-positive puncta in both neurons and astrocytes, (Fig 1D and 1G–1G’). A population of p62 and ubiquitin assembled into ring-like structures, which appeared more abundant in astrocytes (Fig 1D). These structures resemble those previously reported to form during p62 engagement with ubiquitinated substrates on damaged lysosomes [3,25,26]. Consistent with this interpretation, p62 localization partially overlapped with LAMP1 with LLOMe treatment in both neurons and astrocytes (Fig 1C’). Together, these data demonstrate that lysosomal membrane damage induces recruitment of p62 and ubiquitin to lysosomes in both neurons and astrocytes, with both cell types exhibiting similar responses.

### p62 recruitment to damaged lysosomes depends on ubiquitin in both neurons and astrocytes

Previous studies established that ubiquitination of lysosomal proteins is a key step in recruiting p62 to damaged lysosomes, however, this work was performed mostly in non-neural cell types [3,25,26]. To define the role of ubiquitin in p62 recruitment during lysosomal damage in neurons and astrocytes, we pharmacologically reduced ubiquitination using the E1 enzyme inhibitor TAK-243. Substrate ubiquitination proceeds through an enzymatic cascade involving E1 ubiquitin-activating enzymes, E2 ubiquitin-conjugating enzymes, and E3 ubiquitin ligases, which attach ubiquitin primarily to lysine residues on target proteins. TAK-243 binds to the ATPase pocket of the E1 enzyme, thereby preventing transfer of ubiquitin to E2 enzymes and blocking downstream substrate ubiquitination [57].

To ensure that substrates were not sequestered within organelles (e.g., autophagosomes or lysosomes) and thus shielded from the TAK-243 treatment, cocultures were pretreated with TAK-243 prior to induction of lysosomal damage. Neuron-astrocyte cocultures were treated for 1.5 hours with 1 µM TAK-243 or an equivalent volume of DMSO as solvent control (Fig 2A). Lysosomal damage was then induced by adding 1 mM LLOMe for 1 hour in the continued presence of 1 µM TAK-243 or DMSO, followed by immunostaining for GFP (LC3; labels GFP-LC3 transgenic astrocytes), p62, and ubiquitin (Fig 2A).

In DMSO-pretreated controls, LLOMe triggered the formation of p62- and ubiquitin-positive puncta and ring-like structures in both neurons and astrocytes (Fig 2B), consistent with the results shown in Fig 1. Ubiquitin-positive rings co-labeled with p62 were particularly prominent in astrocytes (Fig 2B), matching structures previously linked to lysosomal damage. Pretreatment with TAK-243 significantly reduced ubiquitin-positive structures induced by LLOMe in both neurons and astrocytes (Fig 2B, 2D and 2D’), confirming effective inhibition of ubiquitination and validating antibody specificity. TAK-243 pretreatment also decreased the formation of p62-positive puncta in both cell types (Fig 2B–2C’), indicating that ubiquitin likely contributes to the formation of p62 structures upon lysosomal damage in neurons and astrocytes.

Given that LC3 is a ubiquitin-like protein, we verified that TAK-243 treatment did not impair the conjugation of LC3 to lipids. Indeed, LLOMe effectively induced the formation of LC3-positive structures in astrocytes in the presence of TAK-243, indicating that LC3 conjugation was not impeded by TAK-243 (Fig 2B). Together, these results demonstrate that neurons and astrocytes similarly depend on ubiquitin for recruitment of p62 to damaged lysosomes. This finding demonstrates that mechanisms previously defined mostly in immortalized cell lines are conserved in neurons and astrocytes. Furthermore, these data validate our experimental tools for probing ubiquitin-dependent regulation of p62 under other stress paradigms.

### Neurons and astrocytes distinctly regulate p62-ubiquitin conjugates during metabolic stress

We next investigated whether neurons and astrocytes distinctly regulate p62-ubiquitin conjugates in response to metabolic stress. Thus, we examined the regulation of these conjugates under nutrient deprivation. During nutrient deprivation, autophagy is activated to degrade cellular components to supply amino acids as biosynthetic fuel for maintaining essential functions [32,58,59]. Nutrient deprivation can also increase p62-ubiquitin conjugates in HEK293 cells [24], but whether this result extends to neurons and astrocytes is unknown. Cocultures were starved in Earle’s Balanced Salt Solution (EBSS), a nutrient deprivation model lacking amino acids with reduced glucose compared to standard coculture medium (5.6 mM D-glucose versus 25 mM in standard coculture media). Cocultures were exposed to EBSS for 30 minutes to model short-term metabolic stress. We previously established that 30 minutes of EBSS treatment is sufficient to starve monocultured neurons and astrocytes, as indicated by reduced mTOR signaling [32].

After starvation, cocultures were immunostained for p62, ubiquitin, and GFP (LC3; astrocytes were identified by GFP-LC3 expression). We noted that starvation caused the formation of ubiquitin-positive puncta within the nucleus in neurons. To ensure that our quantification only captured ubiquitin structures linked to quality control in the cytoplasm, we excluded the area of the nucleus and quantified only cytoplasmic puncta within the soma. Thirty minutes of EBSS treatment significantly increased p62-positive puncta in both neurons and astrocytes relative to fed controls (Fig 3A–3B’). This clustering of p62 is consistent with engagement of cargoes that might be routed for autophagic degradation in response to metabolic stress. Strikingly, however, EBSS treatment increased ubiquitin-positive puncta more strongly in neurons than in astrocytes (Fig 3A, 3C–3C’). Furthermore, the degree of colocalization between p62 and ubiquitin puncta also increased more prominently in neurons (Fig 3A, Fig 3D–3D’). This attenuated ubiquitin response in astrocytes contrasted with the lysosomal damage paradigm, where both neurons and astrocytes exhibited a robust increase in p62 and ubiquitin colocalization (Figs 1 and 2).

Since astrocytes exhibited an attenuated ubiquitin response to EBSS treatment, we wanted to confirm that astrocytes were effectively starved. Thus, we quantified GFP-LC3-positive puncta and overlap between LC3 and p62. Thirty minutes of EBSS treatment significantly increased both measurements (Figs 3A and S2B and S2C). These findings confirm that astrocytes experienced nutrient deprivation and activated autophagy accordingly.

Given that short-term starvation produced distinct responses in neurons and astrocytes, we next examined whether prolonged nutrient deprivation would elicit similar differences in p62-ubiquitin regulation across these cell types. Thus, we starved cocultures in EBSS for 4 hours to model long-term metabolic stress. Consistent with short-term nutrient depletion, 4 hours of EBSS increased p62-positive puncta in both neurons and astrocytes (S3A–S3B’ Fig). However, ubiquitin-positive puncta again accumulated more prominently in neurons (S3A, S3C–S3C’ Fig), and colocalization between p62 and ubiquitin puncta remained greater in neurons than in astrocytes (S3A, S3D–S3D’ Fig). Extended starvation also increased GFP(LC3) puncta and GFP(LC3)-p62 overlap in astrocytes (S2D–S2E Fig), confirming activation of autophagy. Together, these findings indicate that both short- and long-term nutrient deprivation preferentially induce formation of p62-ubiquitin conjugates in neurons relative to astrocytes. Thus, neurons and astrocytes can diverge in how they deploy p62 and ubiquitin in a manner that depends on the specific stress.

### p62 clustering during metabolic stress depends more strongly on ubiquitin in neurons than in astrocytes

To define the role of ubiquitin in regulating p62 during metabolic stress, we reduced ubiquitination using TAK-243, as described in Fig 2. Cocultures were pretreated for 1.5 hours with 1 µM TAK-243 or with DMSO as a solvent control, then subjected to EBSS starvation for 30 minutes in the continued presence of the inhibitor or control (Fig 4A). To distinguish cell types, neurons or astrocytes derived from GFP-LC3 transgenic mice were cocultured with their non-transgenic counterpart (Figs 4B and S1C). Consistent with earlier results, EBSS treatment in the DMSO pretreatment group increased p62 puncta in both neurons and astrocytes but enriched ubiquitin puncta preferentially in neurons (Fig 4B).

Pretreatment with TAK-243 effectively reduced ubiquitin-positive structures in both neurons and astrocytes (Fig 4B, 4D–4D’). Interestingly, inhibition of ubiquitination selectively decreased p62 puncta density in neurons but not in astrocytes (Fig 4B, 4C–4C’). In astrocytes, TAK-243 treatment instead increased the total area occupied by p62 puncta (Fig 4B and 4C’). This increase in p62 area was not due to a change in the number of p62 puncta (S4A Fig), but might be due to an increase in the mean size of p62 puncta per cell (S4B Fig). The percentage of p62 puncta colocalized with GFP-LC3 was unaltered with TAK-243 in astrocytes (S2G Fig), indicating continued routing of p62 to autophagosomes. Moreover, TAK-243 did not reduce GFP-LC3 puncta in astrocytes during starvation (S2F Fig), indicating that autophagosome formation itself was not impaired by reduced ubiquitination.

Together, these results demonstrate that neurons depend more strongly on ubiquitin to cluster p62 during metabolic stress than astrocytes. Hence, we reveal that distinct mechanisms underlie how different neural cell types use ubiquitin to regulate p62. Collectively, our work establishes a foundation for examining how differential regulation of p62-ubiquitin conjugates in neurons and astrocytes may influence selective vulnerability and proteostasis failure in neurodegenerative disease.

## Discussion

Here, we examined how neurons and astrocytes differentially regulate p62-ubiquitin conjugates in response to two distinct forms of cellular stress: lysosomal membrane damage and metabolic stress induced by nutrient deprivation. To define these cell-type-specific responses, we used a neuron-astrocyte coculture system that reproduces key morphological, proteomic, and functional features of neuron-astrocyte interactions observed *in vivo*. Lysosomal membrane damage triggered the formation of punctate and ring-like structures positive for p62 and ubiquitin in both neurons and astrocytes (Fig 1). The formation of these p62-positive structures was dependent on ubiquitin in both cell types (Fig 2). Thus, neurons and astrocytes respond similarly to lysosomal membrane injury by engaging p62-ubiquitin conjugates, consistent with mechanisms described in immortalized cell lines where p62 functions in lysophagy.

In contrast, metabolic stress induced by amino acid deprivation and reduced glucose elicited divergent responses between neurons and astrocytes. Both short-term (30 minutes) and long-term starvation (4 hours) increased p62-ubiquitin structures more prominently in neurons than in astrocytes (Fig 3 and S3). Neurons also showed a greater reliance on ubiquitin to form p62 puncta during metabolic stress (Fig 4). Even when ubiquitination was reduced by E1 inhibition, astrocytes efficiently delivered p62 to autophagosomes (S2G Fig), and autophagosome formation itself was not impaired (S2F Fig). Collectively, these results demonstrate that neurons and astrocytes differentially regulate p62 and ubiquitin in a stress-specific manner, highlighting context- and cell-type-specific control of proteostasis pathways.

One limitation of using an E1 inhibitor is that some of the effects on p62 may be indirect due to a global disruption of ubiquitin-dependent processes. However, given that p62 is a ubiquitin-binding protein, it is likely that the observed effects are largely due to a direct impact on p62 engagement with ubiquitinated substrates. Follow-up studies could target specific E3 ubiquitin ligases modifying specific cargoes. For example, multiple E3 ubiquitin ligase complexes have been implicated in lysophagy including SCF^FBXO3^ [60], SCF^FBXO27^ [61], CUL4A-DDB1-WDFY1 [62], ITCH [63], and TRIM16 [64] (reviewed in [65]), as well as the E2 ubiquitin-conjugating enzyme UBE2QL1 [26]. Genetic approaches that disrupt the activity of these E3 ligases, or the capacity of p62 to bind ubiquitin, would be an alternative strategy to interrogate p62 engagement with ubiquitinated substrates.

A potential model emerging from these findings is that neurons and astrocytes may target distinct cargoes for degradation under metabolic stress. The strong dependence on ubiquitin in neurons suggests that they may be more selective in the cargoes routed for degradation during nutrient deprivation. In contrast, the relatively muted ubiquitin response in starved astrocytes implies a greater reliance on bulk autophagy, which can non-selectively engulf cytoplasmic material. These observations raise the possibility that neurons may engage more selective quality control mechanisms, whereas astrocytes may favor generalized clearance pathways to preserve metabolic stability.

Why would neurons and astrocytes exhibit different proteostatic responses to metabolic stress? These cell type differences may be due, in part, to their distinct yet interconnected metabolic programs. Astrocytes are highly glycolytic, whereas neurons depend primarily on oxidative phosphorylation for ATP production [66–70]. Metabolic coupling between neurons and astrocytes allows synaptic activity to stimulate glycolysis in astrocytes, leading to lactate production and export to neurons, where it serves as an energy substrate and supports long-term memory formation [66,70–73]. Astrocytes also release ATP, which modulates neuronal activity [74–77], and lysosomes enriched in ATP have been proposed as a source of extracellular ATP released through lysosomal exocytosis [78,79]. Extracellular ATP can be metabolized to adenosine which activates neuronal adenosine receptors to modulate neuronal activity [74–77]. In total, these different metabolic roles may require astrocytes to deploy p62-dependent homeostatic mechanisms differently than neurons. In this way, astrocytes may also use autophagy to mobilize resources to support metabolic and synaptic demands of neurons. To untangle the role of neuron-astrocyte coupling in the cell stress response, future studies could compare the effects of metabolic stress on each cell type when cultured together versus individually. Previously we found that monocultured astrocytes induce autophagy more robustly than monocultured neurons in response to starvation as measured with GFP-LC3 [32]. Additionally, experiments could compare stress responses at various durations in coculture that would capture a spectrum of synaptic maturity. In this way, one would begin to dissect how these cell-type-specific responses are affected by the degree of synaptic connectivity.

This cooperative metabolic relationship extends to lipid and redox homeostasis, which may also impose cell-type-selective mechanisms of quality control that implicate p62. During periods of high synaptic activity, toxic peroxidated fatty acids generated in neurons are transferred to astrocytes, stored in lipid droplets, and subsequently catabolized [80]. This process triggers an astrocytic transcriptional response that mitigates oxidative stress [80]. Additionally, synaptic activity or oxidative stress can activate the transcription factor NRF2 in astrocytes, which drives expression of antioxidant genes [81,82]. This NRF2 response exerts neuroprotective effects, potentially through transfer of antioxidants that alleviates oxidative stress in neurons [82–86].

Intriguingly, beyond roles in selective autophagy, p62 also promotes antioxidant signaling. p62 activates NRF2 by binding to KEAP1 [15,87], an adaptor of the Cullin-3-type ubiquitin ligase complex that targets NRF2 for proteasomal degradation [88–90]. By sequestering KEAP1, p62 disrupts the KEAP1-NRF2 interaction, allowing NRF2 to translocate to the nucleus and induce transcription of antioxidant genes [15,87]. Oxidative stress promotes phosphorylation of p62 within the KEAP1-interacting region, which enhances affinity for KEAP1 [17]. Notably, p62 is also a transcriptional target of NRF2, forming a positive feedback loop that amplifies the antioxidant response [91]. Various forms of nutrient deprivation, including amino acid, serum, or glucose starvation, are known to elevate reactive oxygen species (ROS) and increase oxidative stress [92–94]. Moreover, a recent study demonstrates that nutrient deprivation increases p62 phosphorylation and activates NRF2-dependent antioxidant signaling [95]. Thus, metabolic stress may engage p62 as an autophagy receptor and as a modulator of redox homeostasis. Since NRF2 signaling has been reported to be enriched in astrocytes [81,82], the p62 structures that we observed in astrocytes under metabolic stress may participate in the KEAP1-NRF2 pathway to promote antioxidant defense. In this model, metabolic stress could elicit distinct functional outcomes for p62 in neurons and astrocytes, favoring selective autophagy in neurons and antioxidant signaling in astrocytes. Furthermore, ALS-FTLD-linked mutations in p62 disrupt both selective autophagy and NRF2-mediated antioxidant pathways [96]. Such disease-associated variants may therefore impair these cell-type-specific functions of p62, creating distinct neuronal and astrocytic vulnerabilities that collectively drive neurodegenerative progression. Together, these observations highlight how stress-dependent regulation of p62 may influence selective vulnerability in ALS and related disorders. Future studies will be needed to test this model and define how p62 coordinates divergent stress responses across cell types.

Another possible mechanism for the cell-type-specific divergence in p62 regulation is that p62 structures formed in astrocytes during metabolic stress may correspond to stress granules. Stress granules assemble in response to diverse stress conditions, including nutrient deprivation, particularly under reduced or depleted glucose levels [97–101]. Several studies have shown that p62 associates with stress granules formed during arsenite-induced oxidative stress and promotes their clearance through autophagy [11,12]. Notably, the association of p62 with stress granules occurs through multiple mechanisms, including those that may be distinct from ubiquitination. Chitiprolu et al. reported that ubiquitin is not enriched in stress granules [11], and proposed that p62 is recruited through interactions with arginine-dimethylated proteins, such as FUS, which are abundant in these structures [11]. In addition, Jeon et al. found that p62-mediated clearance of stress granules may involve interactions with NS1 binding protein (NS1-BP); NS1-BP binds to the UBA domain of p62 [12]. In this context, NS1-BP suppresses ubiquitination of p62 which may stabilize p62 to promote autophagic removal of stress granules [12]. Interestingly, arsenite-induced oxidative stress leads to faster stress granule formation and disassembly in astrocytes than in neurons [102]. These observations raise the possibility that under metabolic stress, p62 may play dual roles in astrocytes, supporting antioxidant defense through the KEAP1-NRF2 pathway and modulating stress granule dynamics.

Our study establishes a framework for understanding how neurons and astrocytes differentially deploy p62 in response to distinct forms of cellular stress. Future investigations will need to define how p62 is balanced between autophagy and the ubiquitin-proteasome system [1,13]. Primary astrocytes have been reported to exhibit higher proteasomal activity than primary neurons [103], suggesting that these cell types may differ in how proteostatic resources are allocated [33]. Defining how this balance shifts under different stress conditions will be essential for understanding how neurons and astrocytes preserve homeostasis and adapt to metabolic and proteotoxic challenges. More broadly, delineating cell-type-specific p62 stress responses may provide insights into cell type vulnerabilities in neurodegenerative disease.

## Supporting information

S1 Fig Method for coculturing neurons and astrocytes. **(A)** Cortical mouse neurons (~96% pure) are isolated from either GFP-LC3 transgenic (Tg) embryos or non-transgenic (nTg) embryos at embryonic day 15 (E15) and plated onto glass coverslips. **(B)** Cortical glia enriched for astrocytes are isolated from P0-P1 pups of the opposing genotype and **(A)** plated onto neurons that are at DIV4–5. Neurons and astrocytes are then cocultured for 7–8 days (neurons are a total age of DIV11–13 in culture) before performing experiments. **(C)** Cell-type-specific genotype combinations used in this study are denoted within each respective figure.
(EPS)

S2 Fig Quantification of GFP(LC3) puncta area in transgenic astrocytes under different treatment paradigms. **(A)** The graph in A corresponds to the experiment and primary data in Fig 1D. Co-cultures of GFP-LC3 transgenic astrocytes and non-transgenic neurons were treated for 1 hr with 1 mM LLOMe to induce lysosomal damage, or an equivalent volume of DMSO solvent as a control. Quantification of total area occupied by GFP(LC3)-positive puncta normalized to soma area in astrocytes. Horizontal bars represent the means of the biological replicates ± SEM; shown is the p-value from a LME model; N = 32 astrocytes from 3 independent experiments; 7–8 DIV of coculture. **(B-E)** The graphs in B-C correspond to the experiment and primary data in Fig 3A. The graphs in D-E correspond to the experiment and primary data in Fig S3A. Cocultures of GFP-LC3 transgenic astrocytes and non-transgenic neurons were treated with EBSS (or regular media as a fed control) for 30 minutes **(B-C)** or 4 hours **(D-E)**. Quantification of area occupied by GFP(LC3)-positive puncta per cell normalized to cytoplasmic soma area of each respective cell **(B, D)**, and colocalization area between p62 and GFP(LC3) puncta normalized to cytoplasmic soma area of each respective cell **(C, E)**. **(B-C)** Horizontal bars represent the means of the biological replicates ± SEM; shown are p-values from a LME model; N = 27–30 astrocytes **(B)** or N = 26–27 astrocytes **(C)** from 3 independent experiments; 7 DIV of coculture. **(D-E)** Horizontal bars represent the means of the biological replicates ± SEM; shown are p-values from a LME model; N = 31–35 astrocytes **(D)** or N = 27–32 astrocytes **(E)** from 3 independent experiments; 7–8 DIV of coculture. **(F-G)** The graphs in F-G correspond to the experiment and primary data in Fig 4A, B. Cocultures of GFP-LC3 transgenic astrocytes with non-transgenic neurons were pretreated for 1.5 hr with 1 µM TAK-243 to inhibit the E1 ubiquitin-activating enzyme or an equivalent volume of DMSO solvent as a control, then treated for 30 minutes with EBSS in the continued presence of 1 µM TAK-243 or DMSO. Quantification of total area occupied by GFP(LC3)-positive puncta normalized to cytoplasmic soma area **(F)** or percentage of p62-positive puncta that colocalize with GFP(LC3)-positive puncta normalized to cytoplasmic soma area **(G)**. Horizontal bars represent the means of the biological replicates ± SEM; shown are the p-values from a LME model; N = 33–34 astrocytes **(F)** or N = 32–33 astrocytes **(G)** from 3 independent experiments; 7–8 DIV of coculture.
(EPS)

S3 Fig Long-term metabolic stress preferentially increases p62-ubiquitin structures in neurons as compared to astrocytes. **(A)** Cocultures of neurons and astrocytes were treated for 4 hours in EBSS (or regular media as a fed control) and then immunostained for GFP (LC3; labels GFP-LC3 transgenic astrocytes), p62, and ubiquitin; nuclei (Nuc) were labeled with Hoechst. Shown are maximum projections of z-stacks. Individual images are shown in grayscale; images for GFP(LC3), p62, and ubiquitin are grayscale-matched within a protein marker, across treatment conditions. Filled yellow arrowheads indicate colocalization between p62 puncta and ubiquitin puncta. Empty yellow arrowheads indicate p62 puncta with no ubiquitin puncta correlate. Neurons are circled in orange and astrocytes are circled in purple. Scale bar, 10 µm. **(B-B’)** Quantification of total area occupied by p62-positive puncta normalized to cytoplasmic soma area for neurons **(B)** or astrocytes **(B’)**. Horizontal bars represent the means of the biological replicates ± SEM; shown are the p-values from a LME model; N = 31–36 neurons and N = 30–32 astrocytes from 3 independent experiments; 7–8 DIV of coculture. **(C-C’)** Quantification of area occupied by ubiquitin-positive puncta normalized to cytoplasmic soma area for neurons **(C)** or astrocytes **(C’)**. Horizontal bars represent the means of the biological replicates ± SEM; shown are the p-values from a LME model; N = 32–33 neurons and N = 24–31 astrocytes from 3 independent experiments; 7–8 DIV of coculture. **(D-D’)** Quantification of total area occupied by p62-positive and ubiquitin-positive puncta normalized to cytoplasmic soma area for neurons **(D)** or astrocytes **(D’)**. Horizontal bars represent the means of the biological replicates ± SEM; shown are the p-values from a LME model; N = 30–31 neurons and N = 23–26 astrocytes from 3 independent experiments; 7–8 DIV of coculture.
(EPS)

S4 Fig E1 inhibition during metabolic stress increases p62 puncta size, but not p62 puncta number, in astrocytes. Graphs in Fig S4 correspond to the experiment and primary data in Fig 4A, B. Cocultures of neurons and astrocytes were pretreated for 1.5 hr with 1 µM TAK-243 to inhibit the E1 ubiquitin-activating enzyme or an equivalent volume of DMSO solvent as a control. Cells were then incubated for 30 minutes in EBSS to induce nutrient deprivation, in the continued presence of 1 µM TAK-243 or DMSO. Cells were immunostained for GFP(LC3), p62, and ubiquitin. Shown is the corresponding quantification of the number of p62 puncta per cell **(A)** and mean p62 puncta size (i.e., total puncta area divided by puncta number for each cell) **(B)** for astrocytes; astrocytes were identified with the GFP-LC3 transgene. Horizontal bars represent the means of the biological replicates ± SEM; shown are the p-values from a LME model; N = 32–34 astrocytes **(A-B)** from 3 independent experiments; 7–8 DIV of coculture.
(EPS)

## References

[1] Kumar AV, Mills J, Lapierre LR. Selective autophagy receptor p62/SQSTM1, a pivotal player in stress and aging. Front Cell Dev Biol. 2022;10:793328. doi: 10.3389/fcell.2022.793328 35237597 PMC8883344

[2] Pankiv S, Clausen TH, Lamark T, Brech A, Bruun J-A, Outzen H, et al. p62/SQSTM1 binds directly to Atg8/LC3 to facilitate degradation of ubiquitinated protein aggregates by autophagy. J Biol Chem. 2007;282(33):24131–45. doi: 10.1074/jbc.M702824200 17580304

[3] Gallagher ER, Holzbaur ELF. The selective autophagy adaptor p62/SQSTM1 forms phase condensates regulated by HSP27 that facilitate the clearance of damaged lysosomes via lysophagy. Cell Rep. 2023;42(2):112037. doi: 10.1016/j.celrep.2023.112037 36701233 PMC10366342

[4] Fujioka Y, Alam JM, Noshiro D, Mouri K, Ando T, Okada Y, et al. Phase separation organizes the site of autophagosome formation. Nature. 2020;578(7794):301–5. doi: 10.1038/s41586-020-1977-6 32025038

[5] Kageyama S, Gudmundsson SR, Sou Y-S, Ichimura Y, Tamura N, Kazuno S, et al. p62/SQSTM1-droplet serves as a platform for autophagosome formation and anti-oxidative stress response. Nat Commun. 2021;12(1):16. doi: 10.1038/s41467-020-20185-1 33397898 PMC7782522

[6] Sánchez-Martín P, Sou Y-S, Kageyama S, Koike M, Waguri S, Komatsu M. NBR1-mediated p62-liquid droplets enhance the Keap1-Nrf2 system. EMBO Rep. 2020;21(3):e48902. doi: 10.15252/embr.201948902 31916398 PMC7054683

[7] Wurzer B, Zaffagnini G, Fracchiolla D, Turco E, Abert C, Romanov J, et al. Oligomerization of p62 allows for selection of ubiquitinated cargo and isolation membrane during selective autophagy. Elife. 2015;4:e08941. doi: 10.7554/eLife.08941 26413874 PMC4684078

[8] Zaffagnini G, Savova A, Danieli A, Romanov J, Tremel S, Ebner M, et al. p62 filaments capture and present ubiquitinated cargos for autophagy. EMBO J. 2018;37(5):e98308. doi: 10.15252/embj.201798308 29343546 PMC5830917

[9] Geisler S, Holmström KM, Skujat D, Fiesel FC, Rothfuss OC, Kahle PJ, et al. PINK1/Parkin-mediated mitophagy is dependent on VDAC1 and p62/SQSTM1. Nat Cell Biol. 2010;12(2):119–31. doi: 10.1038/ncb2012 20098416

[10] Narendra D, Kane LA, Hauser DN, Fearnley IM, Youle RJ. p62/SQSTM1 is required for Parkin-induced mitochondrial clustering but not mitophagy; VDAC1 is dispensable for both. Autophagy. 2010;6(8):1090–106. doi: 10.4161/auto.6.8.13426 20890124 PMC3359490

[11] Chitiprolu M, Jagow C, Tremblay V, Bondy-Chorney E, Paris G, Savard A, et al. A complex of C9ORF72 and p62 uses arginine methylation to eliminate stress granules by autophagy. Nat Commun. 2018;9(1):2794. doi: 10.1038/s41467-018-05273-7 30022074 PMC6052026

[12] Jeon P, Ham H-J, Choi H, Park S, Jang J-W, Park S-W, et al. NS1 binding protein regulates stress granule dynamics and clearance by inhibiting p62 ubiquitination. Nat Commun. 2024;15(1):10925. doi: 10.1038/s41467-024-55446-w 39738171 PMC11686067

[13] Seibenhener ML, Babu JR, Geetha T, Wong HC, Krishna NR, Wooten MW. Sequestosome 1/p62 is a polyubiquitin chain binding protein involved in ubiquitin proteasome degradation. Mol Cell Biol. 2004;24(18):8055–68. doi: 10.1128/MCB.24.18.8055-8068.2004 15340068 PMC515032

[14] Pankiv S, Lamark T, Bruun J-A, Øvervatn A, Bjørkøy G, Johansen T. Nucleocytoplasmic shuttling of p62/SQSTM1 and its role in recruitment of nuclear polyubiquitinated proteins to promyelocytic leukemia bodies. J Biol Chem. 2010;285(8):5941–53. doi: 10.1074/jbc.M109.039925 20018885 PMC2820819

[15] Komatsu M, Kurokawa H, Waguri S, Taguchi K, Kobayashi A, Ichimura Y, et al. The selective autophagy substrate p62 activates the stress responsive transcription factor Nrf2 through inactivation of Keap1. Nat Cell Biol. 2010;12(3):213–23. doi: 10.1038/ncb2021 20173742

[16] Lau A, Zheng Y, Tao S, Wang H, Whitman SA, White E, et al. Arsenic inhibits autophagic flux, activating the Nrf2-Keap1 pathway in a p62-dependent manner. Mol Cell Biol. 2013;33(12):2436–46. doi: 10.1128/MCB.01748-12 23589329 PMC3700105

[17] Ichimura Y, Waguri S, Sou Y-S, Kageyama S, Hasegawa J, Ishimura R, et al. Phosphorylation of p62 activates the Keap1-Nrf2 pathway during selective autophagy. Mol Cell. 2013;51(5):618–31. doi: 10.1016/j.molcel.2013.08.003 24011591

[18] Sanz L, Sanchez P, Lallena MJ, Diaz-Meco MT, Moscat J. The interaction of p62 with RIP links the atypical PKCs to NF-kappaB activation. EMBO J. 1999;18(11):3044–53. doi: 10.1093/emboj/18.11.3044 10356400 PMC1171386

[19] Wooten MW, Geetha T, Seibenhener ML, Babu JR, Diaz-Meco MT, Moscat J. The p62 scaffold regulates nerve growth factor-induced NF-kappaB activation by influencing TRAF6 polyubiquitination. J Biol Chem. 2005;280(42):35625–9. doi: 10.1074/jbc.C500237200 16079148

[20] Zotti T, Scudiero I, Settembre P, Ferravante A, Mazzone P, D’Andrea L, et al. TRAF6-mediated ubiquitination of NEMO requires p62/sequestosome-1. Mol Immunol. 2014;58(1):27–31. doi: 10.1016/j.molimm.2013.10.015 24270048 PMC3909464

[21] Duran A, Amanchy R, Linares JF, Joshi J, Abu-Baker S, Porollo A, et al. p62 is a key regulator of nutrient sensing in the mTORC1 pathway. Mol Cell. 2011;44(1):134–46. doi: 10.1016/j.molcel.2011.06.038 21981924 PMC3190169

[22] Linares JF, Duran A, Yajima T, Pasparakis M, Moscat J, Diaz-Meco MT. K63 polyubiquitination and activation of mTOR by the p62-TRAF6 complex in nutrient-activated cells. Mol Cell. 2013;51(3):283–96. doi: 10.1016/j.molcel.2013.06.020 23911927 PMC3971544

[23] Linares JF, Duran A, Reina-Campos M, Aza-Blanc P, Campos A, Moscat J, et al. Amino acid activation of mTORC1 by a PB1-domain-driven kinase complex cascade. Cell Rep. 2015;12(8):1339–52. doi: 10.1016/j.celrep.2015.07.045 26279575 PMC4551582

[24] You Z, Jiang W-X, Qin L-Y, Gong Z, Wan W, Li J, et al. Requirement for p62 acetylation in the aggregation of ubiquitylated proteins under nutrient stress. Nat Commun. 2019;10(1):5792. doi: 10.1038/s41467-019-13718-w 31857589 PMC6923396

[25] Papadopoulos C, Kirchner P, Bug M, Grum D, Koerver L, Schulze N, et al. VCP/p97 cooperates with YOD1, UBXD1 and PLAA to drive clearance of ruptured lysosomes by autophagy. EMBO J. 2017;36(2):135–50. doi: 10.15252/embj.201695148 27753622 PMC5242375

[26] Koerver L, Papadopoulos C, Liu B, Kravic B, Rota G, Brecht L, et al. The ubiquitin-conjugating enzyme UBE2QL1 coordinates lysophagy in response to endolysosomal damage. EMBO Rep. 2019;20(10):e48014. doi: 10.15252/embr.201948014 31432621 PMC6776906

[27] Chen Y, Zheng Z-Z, Chen X, Huang R, Yang Y, Yuan L, et al. SQSTM1 mutations in Han Chinese populations with sporadic amyotrophic lateral sclerosis. Neurobiol Aging. 2014;35(3):726.e7-9. doi: 10.1016/j.neurobiolaging.2013.09.008 24138988

[28] Kwok CT, Morris A, de Belleroche JS. Sequestosome-1 (SQSTM1) sequence variants in ALS cases in the UK: prevalence and coexistence of SQSTM1 mutations in ALS kindred with PDB. Eur J Hum Genet. 2014;22(4):492–6. doi: 10.1038/ejhg.2013.184 23942205 PMC3953910

[29] Teyssou E, Takeda T, Lebon V, Boillée S, Doukouré B, Bataillon G, et al. Mutations in SQSTM1 encoding p62 in amyotrophic lateral sclerosis: genetics and neuropathology. Acta Neuropathol. 2013;125(4):511–22. doi: 10.1007/s00401-013-1090-0 23417734

[30] Hocking LJ, Lucas GJA, Daroszewska A, Mangion J, Olavesen M, Cundy T, et al. Domain-specific mutations in sequestosome 1 (SQSTM1) cause familial and sporadic Paget’s disease. Hum Mol Genet. 2002;11(22):2735–9. doi: 10.1093/hmg/11.22.2735 12374763

[31] Laurin N, Brown JP, Morissette J, Raymond V. Recurrent mutation of the gene encoding sequestosome 1 (SQSTM1/p62) in Paget disease of bone. Am J Hum Genet. 2002;70(6):1582–8. doi: 10.1086/340731 11992264 PMC379146

[32] Kulkarni A, Dong A, Kulkarni VV, Chen J, Laxton O, Anand A, et al. Differential regulation of autophagy during metabolic stress in astrocytes and neurons. Autophagy. 2020;16(9):1651–67. doi: 10.1080/15548627.2019.1703354 31876243 PMC8386594

[33] Yuan R, Hahn Y, Stempel MH, Sidibe DK, Laxton O, Chen J, et al. Proteasomal inhibition preferentially stimulates lysosome activity relative to autophagic flux in primary astrocytes. Autophagy. 2023;19(2):570–96. doi: 10.1080/15548627.2022.2084884 35722992 PMC9851260

[34] Rhoads SN, Dong W, Hsu C-H, Mfulama NR, Yarlagadda R, Ragusa JV, et al. Neurons and astrocytes have distinct organelle signatures and responses to stress. Cell Rep. 2025;44(9):116280. doi: 10.1016/j.celrep.2025.116280 40956669 PMC12631801

[35] Mizushima N, Yamamoto A, Matsui M, Yoshimori T, Ohsumi Y. In vivo analysis of autophagy in response to nutrient starvation using transgenic mice expressing a fluorescent autophagosome marker. Mol Biol Cell. 2004;15(3):1101–11. doi: 10.1091/mbc.e03-09-0704 14699058 PMC363084

[36] Dong A, Kulkarni VV, Maday S. Methods for imaging autophagosome dynamics in primary neurons. Methods Mol Biol. 2019;1880:243–56. doi: 10.1007/978-1-4939-8873-0_16 30610702 PMC9990474

[37] Chen S, Jing Y, Kang X, Yang L, Wang D-L, Zhang W, et al. Histone H2B monoubiquitination is a critical epigenetic switch for the regulation of autophagy. Nucleic Acids Res. 2017;45(3):1144–58. doi: 10.1093/nar/gkw1025 28180298 PMC5388390

[38] Uriarte M, Sen Nkwe N, Tremblay R, Ahmed O, Messmer C, Mashtalir N, et al. Starvation-induced proteasome assemblies in the nucleus link amino acid supply to apoptosis. Nat Commun. 2021;12(1):6984. doi: 10.1038/s41467-021-27306-4 34848715 PMC8633328

[39] Yasuda S, Tsuchiya H, Kaiho A, Guo Q, Ikeuchi K, Endo A, et al. Stress- and ubiquitylation-dependent phase separation of the proteasome. Nature. 2020;578(7794):296–300. doi: 10.1038/s41586-020-1982-9 32025036

[40] Sheng X, Xia Z, Yang H, Hu R. The ubiquitin codes in cellular stress responses. Protein Cell. 2024;15(3):157–90. doi: 10.1093/procel/pwad045 37470788 PMC10903993

[41] Lord SJ, Velle KB, Mullins RD, Fritz-Laylin LK. SuperPlots: Communicating reproducibility and variability in cell biology. J Cell Biol. 2020;219(6):e202001064. doi: 10.1083/jcb.202001064 32346721 PMC7265319

[42] Stogsdill JA, Ramirez J, Liu D, Kim YH, Baldwin KT, Enustun E, et al. Astrocytic neuroligins control astrocyte morphogenesis and synaptogenesis. Nature. 2017;551(7679):192–7. doi: 10.1038/nature24638 29120426 PMC5796651

[43] Markey KM, Saunders JC, Smuts J, von Reyn CR, Garcia ADR. Astrocyte development-More questions than answers. Front Cell Dev Biol. 2023;11:1063843. doi: 10.3389/fcell.2023.1063843 37051466 PMC10083403

[44] Thiele DL, Lipsky PE. Mechanism of L-leucyl-L-leucine methyl ester-mediated killing of cytotoxic lymphocytes: dependence on a lysosomal thiol protease, dipeptidyl peptidase I, that is enriched in these cells. Proc Natl Acad Sci U S A. 1990;87(1):83–7. doi: 10.1073/pnas.87.1.83 2296607 PMC53204

[45] Shima T, Ogura M, Matsuda R, Nakamura S, Jin N, Yoshimori T, et al. The TMEM192-mKeima probe specifically assays lysophagy and reveals its initial steps. J Cell Biol. 2023;222(12):e202204048. doi: 10.1083/jcb.202204048 37801070 PMC10558291

[46] Eapen VV, Swarup S, Hoyer MJ, Paulo JA, Harper JW. Quantitative proteomics reveals the selectivity of ubiquitin-binding autophagy receptors in the turnover of damaged lysosomes by lysophagy. Elife. 2021;10:e72328. doi: 10.7554/eLife.72328 34585663 PMC8523161

[47] Cross J, Durgan J, McEwan DG, Tayler M, Ryan KM, Florey O. Lysosome damage triggers direct ATG8 conjugation and ATG2 engagement via non-canonical autophagy. J Cell Biol. 2023;222(12):e202303078. doi: 10.1083/jcb.202303078 37796195 PMC10561555

[48] Kaur N, Carlsson SR, Lystad AH. Lysosome-associated CASM: from upstream triggers to downstream effector mechanisms. Front Cell Dev Biol. 2025;13:1559125. doi: 10.3389/fcell.2025.1559125 40213394 PMC11983544

[49] Meyer H, Kravic B. The endo-lysosomal damage response. Annu Rev Biochem. 2024;93(1):367–87. doi: 10.1146/annurev-biochem-030222-102505 38594929

[50] Berg S, Kutra D, Kroeger T, Straehle CN, Kausler BX, Haubold C, et al. ilastik: interactive machine learning for (bio)image analysis. Nat Methods. 2019;16(12):1226–32. doi: 10.1038/s41592-019-0582-9 31570887

[51] Smith EM, Chanaday NL, Maday S. Astrocytes mobilize a broader repertoire of lysosomal repair mechanisms than neurons. bioRxiv. 2025;2025:674666. doi: 10.1101/2025.09.07.674666 PMC13487023 42620891

[52] Dantuma NP, Groothuis TAM, Salomons FA, Neefjes J. A dynamic ubiquitin equilibrium couples proteasomal activity to chromatin remodeling. J Cell Biol. 2006;173(1):19–26. doi: 10.1083/jcb.200510071 16606690 PMC2063781

[53] Lie PPY, Yang D-S, Stavrides P, Goulbourne CN, Zheng P, Mohan PS, et al. Post-Golgi carriers, not lysosomes, confer lysosomal properties to pre-degradative organelles in normal and dystrophic axons. Cell Rep. 2021;35(4):109034. doi: 10.1016/j.celrep.2021.109034 33910020 PMC8135226

[54] Cheng X-T, Xie Y-X, Zhou B, Huang N, Farfel-Becker T, Sheng Z-H. Characterization of LAMP1-labeled nondegradative lysosomal and endocytic compartments in neurons. J Cell Biol. 2018;217(9):3127–39. doi: 10.1083/jcb.201711083 29695488 PMC6123004

[55] Yap CC, Digilio L, McMahon LP, Garcia ADR, Winckler B. Degradation of dendritic cargos requires Rab7-dependent transport to somatic lysosomes. J Cell Biol. 2018;217(9):3141–59. doi: 10.1083/jcb.201711039 29907658 PMC6122995

[56] Gowrishankar S, Yuan P, Wu Y, Schrag M, Paradise S, Grutzendler J, et al. Massive accumulation of luminal protease-deficient axonal lysosomes at Alzheimer’s disease amyloid plaques. Proc Natl Acad Sci U S A. 2015;112(28):E3699-708. doi: 10.1073/pnas.1510329112 26124111 PMC4507205

[57] Hyer ML, Milhollen MA, Ciavarri J, Fleming P, Traore T, Sappal D, et al. A small-molecule inhibitor of the ubiquitin activating enzyme for cancer treatment. Nat Med. 2018;24(2):186–93. doi: 10.1038/nm.4474 29334375

[58] Klionsky DJ, Emr SD. Autophagy as a regulated pathway of cellular degradation. Science. 2000;290(5497):1717–21. doi: 10.1126/science.290.5497.1717 11099404 PMC2732363

[59] Shang L, Chen S, Du F, Li S, Zhao L, Wang X. Nutrient starvation elicits an acute autophagic response mediated by Ulk1 dephosphorylation and its subsequent dissociation from AMPK. Proc Natl Acad Sci U S A. 2011;108(12):4788–93. doi: 10.1073/pnas.1100844108 21383122 PMC3064373

[60] Park NY, Jo DS, Yang JY, Bae JE, Kim JB, Kim YH, et al. Activation of lysophagy by a TBK1-SCF(FBXO3)-TMEM192-TAX1BP1 axis in response to lysosomal damage. Nat Commun. 2025;16(1):1109. doi: 10.1038/s41467-025-56294-y 39875384 PMC11775327

[61] Yoshida Y, Yasuda S, Fujita T, Hamasaki M, Murakami A, Kawawaki J, et al. Ubiquitination of exposed glycoproteins by SCFFBXO27 directs damaged lysosomes for autophagy. Proc Natl Acad Sci U S A. 2017;114(32):8574–9. doi: 10.1073/pnas.1702615114 28743755 PMC5559013

[62] Teranishi H, Tabata K, Saeki M, Umemoto T, Hatta T, Otomo T, et al. Identification of CUL4A-DDB1-WDFY1 as an E3 ubiquitin ligase complex involved in initiation of lysophagy. Cell Rep. 2022;40(11):111349. doi: 10.1016/j.celrep.2022.111349 36103833

[63] Gahlot P, Kravic B, Rota G, van den Boom J, Levantovsky S, Schulze N, et al. Lysosomal damage sensing and lysophagy initiation by SPG20-ITCH. Mol Cell. 2024;84(8):1556-1569.e10. doi: 10.1016/j.molcel.2024.02.029 38503285

[64] Chauhan S, Kumar S, Jain A, Ponpuak M, Mudd MH, Kimura T, et al. TRIMs and galectins globally cooperate and TRIM16 and galectin-3 co-direct autophagy in endomembrane damage homeostasis. Dev Cell. 2016;39(1):13–27. doi: 10.1016/j.devcel.2016.08.003 27693506 PMC5104201

[65] Nakamura S, Shima T, Yoshimori T. Molecular mechanisms of the lysosomal damage response and its roles in aging and disease. J Cell Sci. 2025;138(23):jcs264255. doi: 10.1242/jcs.264255 41342389

[66] Bélanger M, Allaman I, Magistretti PJ. Brain energy metabolism: focus on astrocyte-neuron metabolic cooperation. Cell Metab. 2011;14(6):724–38. doi: 10.1016/j.cmet.2011.08.016 22152301

[67] Zheng X, Boyer L, Jin M, Mertens J, Kim Y, Ma L, et al. Metabolic reprogramming during neuronal differentiation from aerobic glycolysis to neuronal oxidative phosphorylation. Elife. 2016;5:e13374. doi: 10.7554/eLife.13374 27282387 PMC4963198

[68] Hall CN, Klein-Flügge MC, Howarth C, Attwell D. Oxidative phosphorylation, not glycolysis, powers presynaptic and postsynaptic mechanisms underlying brain information processing. J Neurosci. 2012;32(26):8940–51. doi: 10.1523/JNEUROSCI.0026-12.2012 22745494 PMC3390246

[69] Pellerin L, Magistretti PJ. Glutamate uptake into astrocytes stimulates aerobic glycolysis: a mechanism coupling neuronal activity to glucose utilization. Proc Natl Acad Sci U S A. 1994;91(22):10625–9. doi: 10.1073/pnas.91.22.10625 7938003 PMC45074

[70] Bonvento G, Bolaños JP. Astrocyte-neuron metabolic cooperation shapes brain activity. Cell Metab. 2021;33(8):1546–64. doi: 10.1016/j.cmet.2021.07.006 34348099

[71] Hasel P, Dando O, Jiwaji Z, Baxter P, Todd AC, Heron S, et al. Neurons and neuronal activity control gene expression in astrocytes to regulate their development and metabolism. Nat Commun. 2017;8:15132. doi: 10.1038/ncomms15132 28462931 PMC5418577

[72] Muraleedharan R, Gawali MV, Tiwari D, Sukumaran A, Oatman N, Anderson J, et al. AMPK-regulated astrocytic lactate shuttle plays a non-cell-autonomous role in neuronal survival. Cell Rep. 2020;32(9):108092. doi: 10.1016/j.celrep.2020.108092 32877674 PMC7531170

[73] Suzuki A, Stern SA, Bozdagi O, Huntley GW, Walker RH, Magistretti PJ, et al. Astrocyte-neuron lactate transport is required for long-term memory formation. Cell. 2011;144(5):810–23. doi: 10.1016/j.cell.2011.02.018 21376239 PMC3073831

[74] Gordon GRJ, Baimoukhametova DV, Hewitt SA, Rajapaksha WRAKJS, Fisher TE, Bains JS. Norepinephrine triggers release of glial ATP to increase postsynaptic efficacy. Nat Neurosci. 2005;8(8):1078–86. doi: 10.1038/nn1498 15995701

[75] Gourine AV, Kasymov V, Marina N, Tang F, Figueiredo MF, Lane S. Astrocytes control breathing through pH-dependent release of ATP. Science. 2010;329(5991):571–5. doi: 10.1126/science.1190721 20647426 PMC3160742

[76] Lefton KB, Wu Y, Dai Y, Okuda T, Zhang Y, Yen A, et al. Norepinephrine signals through astrocytes to modulate synapses. Science. 2025;388(6748):776–83. doi: 10.1126/science.adq5480 40373122 PMC12309572

[77] Chen AB, Duque M, Rymbek A, Dhanasekar M, Wang VM, Mi X, et al. Norepinephrine changes behavioral state through astroglial purinergic signaling. Science. 2025;388(6748):769–75. doi: 10.1126/science.adq5233 40373133 PMC12265949

[78] Zhang Z, Chen G, Zhou W, Song A, Xu T, Luo Q, et al. Regulated ATP release from astrocytes through lysosome exocytosis. Nat Cell Biol. 2007;9(8):945–53. doi: 10.1038/ncb1620 17618272

[79] Beckel JM, Gómez NM, Lu W, Campagno KE, Nabet B, Albalawi F, et al. Stimulation of TLR3 triggers release of lysosomal ATP in astrocytes and epithelial cells that requires TRPML1 channels. Sci Rep. 2018;8(1):5726. doi: 10.1038/s41598-018-23877-3 29636491 PMC5893592

[80] Ioannou MS, Jackson J, Sheu S-H, Chang C-L, Weigel AV, Liu H, et al. Neuron-astrocyte metabolic coupling protects against activity-induced fatty acid toxicity. Cell. 2019;177(6):1522-1535.e14. doi: 10.1016/j.cell.2019.04.001 31130380

[81] Habas A, Hahn J, Wang X, Margeta M. Neuronal activity regulates astrocytic Nrf2 signaling. Proc Natl Acad Sci U S A. 2013;110(45):18291–6. doi: 10.1073/pnas.1208764110 24145448 PMC3831500

[82] Kraft AD, Johnson DA, Johnson JA. Nuclear factor E2-related factor 2-dependent antioxidant response element activation by tert-butylhydroquinone and sulforaphane occurring preferentially in astrocytes conditions neurons against oxidative insult. J Neurosci. 2004;24(5):1101–12. doi: 10.1523/JNEUROSCI.3817-03.2004 14762128 PMC6793572

[83] Vargas MR, Johnson DA, Sirkis DW, Messing A, Johnson JA. Nrf2 activation in astrocytes protects against neurodegeneration in mouse models of familial amyotrophic lateral sclerosis. J Neurosci. 2008;28(50):13574–81. doi: 10.1523/JNEUROSCI.4099-08.2008 19074031 PMC2866507

[84] Shih AY, Johnson DA, Wong G, Kraft AD, Jiang L, Erb H, et al. Coordinate regulation of glutathione biosynthesis and release by Nrf2-expressing glia potently protects neurons from oxidative stress. J Neurosci. 2003;23(8):3394–406. doi: 10.1523/JNEUROSCI.23-08-03394.2003 12716947 PMC6742304

[85] Dringen R, Pfeiffer B, Hamprecht B. Synthesis of the antioxidant glutathione in neurons: supply by astrocytes of CysGly as precursor for neuronal glutathione. J Neurosci. 1999;19(2):562–9. doi: 10.1523/JNEUROSCI.19-02-00562.1999 9880576 PMC6782200

[86] Sagara JI, Miura K, Bannai S. Maintenance of neuronal glutathione by glial cells. J Neurochem. 1993;61(5):1672–6. doi: 10.1111/j.1471-4159.1993.tb09802.x 8228986

[87] Lau A, Wang X-J, Zhao F, Villeneuve NF, Wu T, Jiang T, et al. A noncanonical mechanism of Nrf2 activation by autophagy deficiency: direct interaction between Keap1 and p62. Mol Cell Biol. 2010;30(13):3275–85. doi: 10.1128/MCB.00248-10 20421418 PMC2897585

[88] Cullinan SB, Gordan JD, Jin J, Harper JW, Diehl JA. The Keap1-BTB protein is an adaptor that bridges Nrf2 to a Cul3-based E3 ligase: oxidative stress sensing by a Cul3-Keap1 ligase. Mol Cell Biol. 2004;24(19):8477–86. doi: 10.1128/MCB.24.19.8477-8486.2004 15367669 PMC516753

[89] Kobayashi A, Kang M-I, Okawa H, Ohtsuji M, Zenke Y, Chiba T, et al. Oxidative stress sensor Keap1 functions as an adaptor for Cul3-based E3 ligase to regulate proteasomal degradation of Nrf2. Mol Cell Biol. 2004;24(16):7130–9. doi: 10.1128/MCB.24.16.7130-7139.2004 15282312 PMC479737

[90] Zhang DD, Lo S-C, Cross JV, Templeton DJ, Hannink M. Keap1 is a redox-regulated substrate adaptor protein for a Cul3-dependent ubiquitin ligase complex. Mol Cell Biol. 2004;24(24):10941–53. doi: 10.1128/MCB.24.24.10941-10953.2004 15572695 PMC533977

[91] Jain A, Lamark T, Sjøttem E, Larsen KB, Awuh JA, Øvervatn A, et al. p62/SQSTM1 is a target gene for transcription factor NRF2 and creates a positive feedback loop by inducing antioxidant response element-driven gene transcription. J Biol Chem. 2010;285(29):22576–91. doi: 10.1074/jbc.M110.118976 20452972 PMC2903417

[92] Chen Y, Azad MB, Gibson SB. Superoxide is the major reactive oxygen species regulating autophagy. Cell Death Differ. 2009;16(7):1040–52. doi: 10.1038/cdd.2009.49 19407826

[93] Li R, Kato H, Fumimoto C, Nakamura Y, Yoshimura K, Minagawa E. Essential amino acid starvation-induced oxidative stress causes DNA damage and apoptosis in murine osteoblast-like cells. Int J Mol Sci. 2023;24(20):15314. doi: 10.3390/ijms242015314 37894999 PMC10607495

[94] Scherz-Shouval R, Shvets E, Fass E, Shorer H, Gil L, Elazar Z. Reactive oxygen species are essential for autophagy and specifically regulate the activity of Atg4. EMBO J. 2007;26(7):1749–60. doi: 10.1038/sj.emboj.7601623 17347651 PMC1847657

[95] Choi E-J, Oh H-T, Lee S-H, Zhang C-S, Li M, Kim S-Y, et al. Metabolic stress induces a double-positive feedback loop between AMPK and SQSTM1/p62 conferring dual activation of AMPK and NFE2L2/NRF2 to synergize antioxidant defense. Autophagy. 2024;20(11):2490–510. doi: 10.1080/15548627.2024.2374692 38953310 PMC11572134

[96] Deng Z, Lim J, Wang Q, Purtell K, Wu S, Palomo GM, et al. ALS-FTLD-linked mutations of SQSTM1/p62 disrupt selective autophagy and NFE2L2/NRF2 anti-oxidative stress pathway. Autophagy. 2020;16(5):917–31. doi: 10.1080/15548627.2019.1644076 31362587 PMC7144840

[97] Reineke LC, Cheema SA, Dubrulle J, Neilson JR. Chronic starvation induces noncanonical pro-death stress granules. J Cell Sci. 2018;131(19):jcs220244. doi: 10.1242/jcs.220244 30185525 PMC6198455

[98] Amen T, Kaganovich D. Stress granules inhibit fatty acid oxidation by modulating mitochondrial permeability. Cell Rep. 2021;35(11):109237. doi: 10.1016/j.celrep.2021.109237 34133922 PMC8220302

[99] Wang T, Tian X, Kim HB, Jang Y, Huang Z, Na CH, et al. Intracellular energy controls dynamics of stress-induced ribonucleoprotein granules. Nat Commun. 2022;13(1):5584. doi: 10.1038/s41467-022-33079-1 36151083 PMC9508253

[100] Jain S, Wheeler JR, Walters RW, Agrawal A, Barsic A, Parker R. ATPase-modulated stress granules contain a diverse proteome and substructure. Cell. 2016;164(3):487–98. doi: 10.1016/j.cell.2015.12.038 26777405 PMC4733397

[101] Faber GP, Gross G, Mualem O, Avivi MY, Waldman Ben-Asher H, Yaron O, et al. Glutamine modulates stress granule formation in cancer cells through core RNA-binding proteins. J Cell Sci. 2025;138(11):jcs263679. doi: 10.1242/jcs.263679 40376753 PMC12188316

[102] Khalfallah Y, Kuta R, Grasmuck C, Prat A, Durham HD, Vande Velde C. TDP-43 regulation of stress granule dynamics in neurodegenerative disease-relevant cell types. Sci Rep. 2018;8(1):7551. doi: 10.1038/s41598-018-25767-0 29765078 PMC5953947

[103] Tydlacka S, Wang C-E, Wang X, Li S, Li X-J. Differential activities of the ubiquitin-proteasome system in neurons versus glia may account for the preferential accumulation of misfolded proteins in neurons. J Neurosci. 2008;28(49):13285–95. doi: 10.1523/JNEUROSCI.4393-08.2008 19052220 PMC2662777

